# Post-discharge outcomes of hospitalized children diagnosed with acute SARS-CoV-2 or MIS-C

**DOI:** 10.3389/fped.2024.1340385

**Published:** 2024-02-12

**Authors:** Ericka L. Fink, Alicia M. Alcamo, Marlina Lovett, Mary Hartman, Cydni Williams, Angela Garcia, Lindsey Rasmussen, Ria Pal, Kurt Drury, Elizabeth MackDiaz, Peter A. Ferrazzano, Leslie Dervan, Brian Appavu, Kellie Snooks, Casey Stulce, Pamela Rubin, Bianca Pate, Nicole Toney, Courtney L. Robertson, Mark S. Wainwright, Juan D. Roa, Michelle E. Schober, Beth S. Slomine

**Affiliations:** ^1^ Department of Critical Care Medicine, UPMC Children’s Hospital of Pittsburgh, Pittsburgh, PA, United States; ^2^ Safar Center for Resuscitation Research, University of Pittsburgh Medical Center, Pittsburgh, PA, United States; ^3^ Department of Anesthesiology and Critical Care Medicine, Children’s Hospital of Philadelphia, University of Pennsylvania, Philadelphia, PA, United States; ^4^ Division of Critical Care Medicine, Department of Pediatrics, Nationwide Children’s Hospital, The Ohio State University College of Medicine, Columbus, OH, United States; ^5^ Division of Pediatric Critical Care Medicine, Seattle Children’s Hospital, University of Washington, Seattle, WA, United States; ^6^ Department of Pediatrics, Pediatric Critical Care and Neurotrauma Recovery Program, Oregon Health & Science University, Portland, OR, United States; ^7^ Division of Pediatric Physical Medicine and Rehabilitation, UPMC Children’s Hospital of Pittsburgh, Pittsburgh, PA, United States; ^8^ Division of Pediatric Critical Care Medicine, Lucile Packard Children’s Hospital, Stanford University, Palo Alto, CA, United States; ^9^ Department of Neurology, Lucile Packard Children’s Hospital, Stanford University, Palo Alto, CA, United States; ^10^ Division of Pediatrics, Comer Children’s Hospital, University of Chicago, Chicago, IL, United States; ^11^ Division of Pediatric Critical Care Medicine, MUSC Shawn Jenkins Children’s Hospital, Charleston, SC, United States; ^12^ Department of Pediatrics, University of Wisconsin, Madison, WI, United States; ^13^ Division of Pediatric Critical Care Medicine, Department of Pediatrics, University of Washington School of Medicine, Seattle, WA, United States; ^14^ Division of Neurology, Barrow Neurological Institute at Phoenix Children’s Hospital, College of Medicine, University of Arizona, Phoenix, AZ, United States; ^15^ Department of Pediatrics, Medical College of Wisconsin, Milwaukee, WI, United States; ^16^ Department of Pediatrics, University of Chicago, Chicago, IL, United States; ^17^ Departments of Anesthesiology and Critical Care Medicine, and Pediatrics, Johns Hopkins Children’s Center, Baltimore, MD, United States; ^18^ Division of Pediatric Neurology, Seattle Children’s Hospital, University of Washington, Seattle, WA, United States; ^19^ Department of Pediatrics, Universidad Nacional de Colombia and Fundación Universitaria de Ciencias de la Salud, Bogotá, Colombia; ^20^ Division of Critical Care, Department of Pediatrics, University of Utah, Salt Lake City, UT, United States; ^21^ Department of Psychiatry and Behavioral Sciences, Kennedy Krieger Institute, Johns Hopkins University School of Medicine, Baltimore, MD, United States

**Keywords:** pediatrics, SARS-CoV-2, child development, patient outcome assessment, post-acute COVID-19 syndrome

## Abstract

**Introduction:**

Hospitalized children diagnosed with SARS-CoV-2-related conditions are at risk for new or persistent symptoms and functional impairments. Our objective was to analyze post-hospital symptoms, healthcare utilization, and outcomes of children previously hospitalized and diagnosed with acute SARS-CoV-2 infection or Multisystem Inflammatory Syndrome in Children (MIS-C).

**Methods:**

Prospective, multicenter electronic survey of parents of children <18 years of age surviving hospitalization from 12 U.S. centers between January 2020 and July 2021. The primary outcome was a parent report of child recovery status at the time of the survey (recovered vs. not recovered). Secondary outcomes included new or persistent symptoms, readmissions, and health-related quality of life. Multivariable backward stepwise logistic regression was performed for the association of patient, disease, laboratory, and treatment variables with recovered status.

**Results:**

The children [*n* = 79; 30 (38.0%) female] with acute SARS-CoV-2 (75.7%) or MIS-C (24.3%) had a median age of 6.5 years (interquartile range 2.0–13.0) and 51 (64.6%) had a preexisting condition. Fifty children (63.3%) required critical care. One-third [23/79 (29.1%)] were not recovered at follow-up [43 (31, 54) months post-discharge]. Admission C-reactive protein levels were higher in children not recovered vs. recovered [5.7 (1.3, 25.1) vs. 1.3 (0.4, 6.3) mg/dl, *p* = 0.02]. At follow-up, 67% overall had new or persistent symptoms. The most common symptoms were fatigue (37%), weakness (25%), and headache (24%), all with frequencies higher in children not recovered. Forty percent had at least one return emergency visit and 24% had a hospital readmission. Recovered status was associated with better total HRQOL [87 (77, 95) vs. 77 (51, 83), *p* = 0.01]. In multivariable analysis, lower admission C-reactive protein [odds ratio 0.90 (95% confidence interval 0.82, 0.99)] and higher admission lymphocyte count [1.001 (1.0002, 1.002)] were associated with recovered status.

**Conclusions:**

Children considered recovered by their parents following hospitalization with SARS-CoV-2-related conditions had less symptom frequency and better HRQOL than those reported as not recovered. Increased inflammation and lower lymphocyte count on hospital admission may help to identify children needing longitudinal, multidisciplinary care.

**Clinical Trial Registration:**

ClinicalTrials.gov (NCT04379089).

## Introduction

Individuals infected by SARS-CoV-2 may have new or long-lasting symptoms, impacting functioning and health-related quality of life (HRQOL) (1). Termed Post-COVID-19 condition by the World Health Organization, overall prevalence is estimated at 10%–20% (>65 million people) (2). Symptoms may be persistent or relapsing and remitting with discernable phenotypes (1, 3–7). Postulated mechanisms of Post-COVID-19 condition include persistent SARS-CoV-2 infection (8, 9), immune dysregulation (10), herpesviruses reactivation (11), autoimmunity (12), endothelial dysfunction (13), and brain network dysfunction (2, 14, 15).

Reports of children with Post-COVID-19 condition demonstrate the need for effective preventative and treatment strategies, and prospective data are scarce (16–18). The diagnosis and prevalence of Post-COVID-19 in children are complicated by socioeconomic and mental health effects of the pandemic, disease-related sequelae, developmental stage, and heterogeneous SARS-CoV-2 manifestations in children [e.g., acute infection vs. post-infectious Multisystem Inflammatory Syndrome in Children (MIS-C)] (19, 20). Further, children with neurologic manifestations and SARS-CoV-2-related conditions may be at increased risk of Post-COVID-19 condition (21).

The Global Consortium Study of Neurologic Dysfunction in COVID-19 (GCS-NeuroCOVID) is a multinational research collaborative initiated in April 2020 to describe the prevalence of and outcomes from neurologic manifestations of SARS-CoV-2 related conditions in hospitalized patients (21–23). The objective of this multicenter, prospective study was to survey a subset of parents about their child and family's health post-hospitalization.

## Materials and methods

### Study design and setting

This is a prospective, multicenter (12 U.S. centers) survey of parents of children from a subset of Tier 1 (3,568 patients from 46 centers in 10 countries) conducted between January 1, 2020, and April 30, 2021 (Supplementary Table S1). Centers screened using locally approved methods including chart review and hospital registries. Local regulatory approval was obtained at each study site. Adult participant and child assent informed consent forms were used per local regulatory guidelines. The University of Pittsburgh Institutional Review Board (STUDY20060012) approved the Data Coordinating Center (DCC) at the University of Pittsburgh to receive and analyze de-identified data. Families were contacted by sites using a variety of IRB-approved methods, thus representing a convenience sample.

#### Inclusion criteria

Parents of children aged <17 years and 6 months at the time of study initiation and enrolled in our Tier 1 cohort who survived hospitalization that included a SARS-CoV-2-related condition as defined previously (21).

#### Exclusion criteria

Non-English-speaking parent.

### Data collection and management

A study manual of operations was created and disseminated to sites. Study procedures were reviewed on DCC-led webinars. A custom, secure REDcap Case Report Form was created for parent input of data (Supplementary Appendix). Data collected included a symptom list (new since discharge or present during hospitalization and continued post-discharge), health care utilization (emergency department or hospital admission), new medications, new referrals, proxy-report child HRQOL [PedsQL (24)], global function [PROMIS Parent Proxy Global Health and Functional Status Scale (FSS) (25, 26)], family [PedsQL Family Impact Module (27)], school and vaccination status, and socioeconomic outcomes of the pandemic [Coronavirus Impact Scale (CIS) (28)] (Supplementary Table S2).

Each parent was assigned a unique study identification number by the DCC and provided by the local site upon informed consent. This gave the family access to complete the study surveys within REDcap and allowed the DCC to merge patient-specific data with post-discharge outcomes. If families were unable to complete the surveys directly in REDcap, local study teams could offer assistance.

The Clinical Research, Investigation, and Systems Modeling of Acute Illness (CRISMA) Center at the University of Pittsburgh managed central data collection, quality, security, and analysis. Centers with a data use agreement in place with the University of Pittsburgh previously submitted Tier 1 patient data during the index hospitalization to the DCC using encrypted email or via upload to a secure cloud (https://www.globus.org/). Race and ethnicity were collected in the parent study to evaluate as risk factors for outcomes given prior literature. Data from Tier 1 were linked with data collected prospectively for this study. Data were stored on a password-protected network, with additional periodic secure offsite backups to the database. Data were screened for missing or implausible information and queries were issued for clarification and adjusted.

### Outcomes

The primary outcome was a parent report of recovery status at the time of follow-up as either recovered or not recovered. Caregivers were asked, “Do you feel your child has fully recovered from his or her COVID-19 or MIS-C related illness?” The possible answers were Yes, No, or Unsure, with No and Unsure combined into “Not Recovered” status for a binary outcome. Secondary outcomes are listed in Supplementary Table S2. Poor HRQOL was defined as a total PedsQL score less than 1 standard deviation below the mean of the sample.

### Statistical analysis

Non-parametric tests were used, and data were presented as median (interquartile range). Comparisons were made between children post-discharge recovered vs. not recovered. Backwards stepwise multivariable logistic regression modeling was performed to identify patient, family, disease, and treatment characteristics associated with recovered status and HRQOL. Covariates included for recovered status that had less than 25% missing and *p* < 0.1 in univariate analysis: sex, any comorbidity, steroid and intravenous immune globulin (IVIG) treatment, acute COVID vs. MIS-C status, lymphocytes and C-reactive protein on admission, and CIS score. Covariates included for HRQOL that had less than 25% missing and *p* < 0.1 in univariate analysis: family impact total score, neurologic comorbidity, remdesivir and IVIG treatment, new impairment at hospital discharge, CIS score, ethnicity, and acute COVID vs. MIS-C status. Missing data were not imputed (thus sample sizes for variables varied). All *p*-values were two-sided, and *p* < 0.05 was considered statistically significant. Data were analyzed using Stata (Version 17, Statacorp LLC, College Station, TX). This article was written according to the STROBE (Strengthening the Reporting of Observational studies in Epidemiology) initiative (29).

## Results

### CONSORT diagram

**Figure 1:**
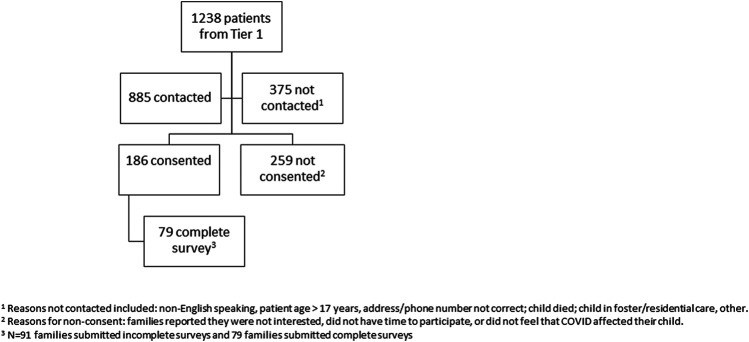
CONSORT diagram.

Among Tier 1 sites that were invited to participate in Tier 2, 12 sites obtained IRB approval, 10 had at least one family complete informed consent, and 9 had at least one family submit survey data (Figure 1). Among 1,238 patients eligible from Tier 1 at participating sites, 885 were contacted by sites, 186 consented, and 79 completed surveys. Follow-up surveys were completed at 530 (382, 652) days from the hospital admission. Children whose caregivers participated in Tier 2 were numerically younger, of less female sex and minority race, and more frequently had a preexisting comorbidity than children whose caregivers who did not participate in Tier 2 (Supplementary Table S1^1^).

**Table 1:** Patient and disease characteristics and outcomes.

Variables, *n* (%) or median (IQR), range	Overall *N* = 79	Not recovered *N* = 23 (29.1%)	Recovered *N* = 56 (70.9%)	*p*-value
Time from hospital admission to follow-up survey completion, days	530 (382, 652)	554 (467, 645)	520 (376, 661)	0.55
Epoch				0.81
January 2020–June 2020	6 (7.6)	1 (4.4)	5 (8.9)	
July 2020–December 2020	31 (39.2)	10 (43.5)	21 (37.5)	
January 2021–July 2021	42 (53.2)	12 (52.2)	30 (53.6)	
Age, years	6.5 (2.0, 13.0), *n* = 78	9.4 (5, 14.0), *n* = 22	6.0 (1.2, 13.0), *n* = 56	0.10
Sex				0.31
Female	30 (38.0)	11 (47.8)	19 (33.9)	
Male	49 (62.0)	12 (52.2)	37 (66.1)	
Race	*N* = 77	*N* = 22	*N* = 55	0.38
Asian	4 (5.2)	0 (0.0)	4 (7.3)	
Black or African American	17 (22.1)	3 (13.6)	14 (25.5)	
White	53 (68.8)	18 (81.8)	35 (63.6)	
Other	3 (3.9)	1 (4.6)	2 (3.6)	
Hispanic Ethnicity	8 (12.1), *n* = 66	2 (10.5), *n* = 19	6 (12.8), *n* = 47	1.00
SARS CoV-2 Diagnosis (from Tier 1)				0.24
Acute SARS-CoV-2	62 (78.5)	16 (69.6)	46 (82.1)	
MIS-C	17 (21.5)	7 (30.4)	10 (17.9)	
Pre-existing condition	51 (64.6)	12 (52.2)	39 (69.6)	0.12
Neurologic	24 (30.4)	7 (30.4)	17 (30.4)	
Non-neurologic	28 (36.4)	6 (28.6)	22 (39.3)	
Cardiovascular	15 (19.0)	1 (4.4)	14 (25.0)	0.06
Respiratory	17 (21.5)	5 (21.7)	12 (21.4)	1.000
Renal or urologic	6 (7.6)	1 (4.4)	5 (8.9)	0.67
Gastrointestinal	11 (14.3), *n* = 77	4 (19.1), *n* = 21	7 (12.5), *n* = 56	0.48
Hematologic or immunologic	8 (10.1)	0 (0.0)	8 (14.3)	0.10
Metabolic	8 (10.1)	0 (0.0)	8 (14.3)	0.10
Congenital or genetic	12 (15.2)	3 (13.0)	9 (16.1)	1.000
Malignancy	0 (0.0)	0 (0.0)	0 (0.0)	–
Premature or neonatal	5 (6.9), *n* = 72	2 (11.1), *n* = 18	3 (5.6), *n* = 54	0.59
Technology dependence	6 (7.6)	1 (4.4)	5 (8.9)	0.67
Transplantation	2 (2.5)	0 (0.0)	2 (3.6)	1.000
Other non-neurologic	7 (9.1), *n* = 77	3 (13.6), *n* = 22	4 (7.3), *n* = 55	0.40
Initial glasgow coma scale score	*N* = 67	*N* = 21	*N* = 46	0.72
13–15	58 (86.6)	18 (85.7)	40 (87.0)	
9–12	4 (6.0)	2 (9.5)	2 (4.4)	
3–8	5 (7.5)	1 (4.8)	4 (8.7)	
Highest level of care				0.31
Intensive care unit	29 (36.7)	6 (26.1)	23 (41.1)	
Ward	50 (63.3)	17 (73.9)	33 (58.9)	
hospital length of stay, days	3 (2, 8)	3 (2, 8)	3 (2, 8)	0.77
Intensive care unit length of stay, days	4 (2, 5), *n* = 29	5 (4, 5), *n* = 6	3 (1, 10), *n* = 23	0.34
Home disposition post-hospital discharge	77 (97.5)	23 (100.0)	54 (96.4)	1.00
Any neurologic manifestation	42 (53.2)	13 (56.5)	29 (51.8)	0.81
Severe neurologic manifestation	24 (30.4)	7 (30.4)	17 (30.4)	1.00
Impairment at hospital discharge^b^	7 (9.1), *n* = 77	1 (4.8), *n* = 21	6 (10.7), *n* = 56	0.67
Initial laboratory testing				
C-reactive protein, mg/dl	3.4 (0.5, 13.1)	5.7 (1.3, 25.1)	1.3 (0.4, 6.3)	0.02
Sodium, mMol/L	137.0 (135.0, 140.0)	137.0 (134.0, 139.0)	137.0 (135.0, 140.0)	0.88
White blood cells (total), per L	8,500.0 (5,600.0, 11,150.0)	8,650.0 (7,050.0, 12,450.0)	8,050.0 (5,200.0, 11,050.0)	0.34
Lymphocytes, gm/dl	1,365.0 (827.0, 2,630.0)	956.0 (592.0, 1,600.0)	1,640.0 (900.0, 2,925.0)	0.07
Hemoglobin, g/dl	12.3 (11.1, 13.4)	12.3 (10.8, 12.9)	12.3 (11.1, 13.7)	0.78
Platelets, per L	219,000.0 (158,000.0, 331,000.0)	207,500.0 (158,500.0, 312,500.0)	219,000.0 (149,000.0, 334,000.0)	0.78
Ferritin, ng/ml	252.0 (136.0, 616.0)	185.0 (72.0, 1,093.0)	261.0 (145.0, 529.0)	0.80
Procalcitonin, ng/ml	0.5 (0.2, 7.3)	0.5 (0.2, 7.9)	0.6 (0.3, 4.5)	0.80
Fibrinogen, mg/dl	463.0 (291.0, 524.0)	502.0 (447.0, 647.0)	336.0 (284.0, 513.0)	0.08
Alanine transaminase, int'l unit/L	24.0 (15.0, 36.0)	24.5 (15.5, 71.5)	24.0 (15.0, 32.0)	0.51
Aspartate transaminase, int'l unit/L	38.0 (25.0, 54.0)	40.0 (24.0, 91.0)	37.5 (25.0, 50.0)	0.59
Prothrombin, seconds	14.7 (13.5, 15.4)	14.7 (13.3, 15.4)	14.6 (13.5, 15.3)	0.73
Partial Thromboplastin Time, seconds	32.0 (29.0, 36.6)	34.0 (29.0, 40.0)	32.0 (29.0, 36.0)	0.48
International Normalized Ratio	1.2 (1.1, 1.3)	1.2 (1.1, 1.3)	1.2 (1.1, 1.3)	0.96
D-dimer, mg/L fibrinogen equivalent units	2.0 (0.7, 3.2)	3.0 (0.7, 3.8)	1.9 (0.8, 2.8)	0.88
Inpatient SARS-CoV-2 related condition treatment^a^				
Any treatment	27 (34.2)	11 (47.8)	16 (28.6)	0.12
Steroids	23 (29.1)	10 (43.5)	13 (23.2)	0.10
Intravenous immune globulin	16 (20.3)	7 (30.4)	9 (16.1)	0.22
Remdesivir	13 (16.5)	4 (17.4)	9 (16.1)	1.00
Monoclonal Ab	1 (1.3)	0 (0.0)	1 (1.8)	1.00

Note that sample size is reported only for variables with sample size <79.

MIS-C, multisystem inflammatory syndrome in children.

^a^ No patient received chloroquine, azithromycin, interleukin-6 inhibitors, plasma exchange, favipiravir, convalescent plasma, lopinavir, or anakinra.

^b^ New impairment at hospital discharge was defined as any baseline to hospital discharge worsening in either the Pediatric Cerebral Performance Category (PCPC) or Functional Status Scale (FSS) score, or discharge PCPC > 1 or FSS > 6.

### Patient and hospital-based characteristics

The median age was 6.5 [interquartile range (IQR) 2.0, 13.0] years and 38.0% were female (Table 1). Of the 51 (64.6%) children with a pre-existing condition, 24 (30.4%) had neurologic conditions. Most (75.5%) children had acute SARS-CoV-2 and 36.7% were admitted to the intensive care unit (Supplementary Table S1). Fewer Hispanic patients were represented in Tier 2 (12.1%) than in Tier 1 (31.1%) and slightly fewer patients in Tier 2 were admitted to the ICU (36.7%) compared with Tier 1 (45.8%). Fewer children had impairment at hospital discharge in Tier 2 (9.1%) vs. Tier 1 (17.0%).

Among families responding to the question, “Do you feel your child has recovered from his or her COVID-19 or MIS-C related illness?”, 70.9% defined their child as recovered and 29.1% as not recovered. Recovered and not recovered groups had similar time to survey, personal characteristics, severity of illness, and frequency of impairment (Table 1). Children who were recovered had decreased median (IQR) initial C-reactive protein (CRP) levels compared to children who were not recovered [1.3 (0.4, 6.3) vs. 5.7 (1.3, 25.1) mg/dl, *p* = 0.02]. Children with MIS-C had higher median (IQR) initial CRP compared with those with acute SARS-CoV-2 [15.0 (5.7, 25.1) vs. 1.0 (0.4, 4.4) mg/dl, *p* < 0.001].

Overall, 34.2% of patients received treatment directed at a SARS-CoV-2 related condition, with no difference by recovered or not recovered status (28.6% vs. 47.8%, *p* = 0.12) (Table 3). Steroids (29.1%) and IVIG (20.3%) were the most common therapies provided in the hospital.

**Table 3:** Post-hospital discharge health care utilization, new consultations, and new medications prescribed.

Variables, *n* (%) or median (IQR), range	Overall *N* = 79	Not recovered *N* = 23 (29.1%)	Recovered *N* = 56 (70.9%)	*p*-value
Emergency department visit	31 (39.2)	13 (56.5)	18 (32.1)	0.07
No. emergency department visits	2 (1, 3), 1–7	2 (1, 3), 1–7	2 (1, 3), 1–5	0.82
Emergency department disposition (first)				0.74
Home	22 (70.8)	10 (76.9)	12 (66.7)	
ICU	2 (6.5)	0 (0.0)	2 (11.1)	
Ward	6 (19.4)	3 (23.1)	3 (16.7)	
Other	1 (3.2)	0 (0.0)	1 (5.7)	
Emergency department disposition (most recent)				0.84
Home	13 (72.2)	5 (71.4)	8 (72.7)	
ICU	1 (5.6)	1 (14.3)	0 (0.0)	
Ward	3 (16.7)	1 (14.3)	2 (18.2)	
Other	1 (5.6)	0 (0.0)	1 (9.1)	
Hospital readmission	19 (24.1)	5 (21.7)	14 (25.0)	1.000
No. hospital readmissions	2 (1, 4), 1–8	4 (3, 5), 1–8	1.5 (1, 3), 1–5	0.060
Follow-up care visits				
Primary care provider	58 (73.4)	22 (95.7)	36 (64.3)	0.004
Cardiology	28 (35.4)	8 (34.8)	20 (35.7)	1.000
Neurology	18 (22.8)	9 (39.1)	9 (16.1)	0.0439
Physical therapy	14 (17.7)	5 (21.7)	9 (16.1)	0.5435
Speech therapy	14 (17.7)	4 (17.4)	10 (17.9)	1.000
Pulmonary	13 (16.5)	7 (30.4)	6 (10.7)	0.05
Occupational therapy	12 (15.2)	5 (21.7)	7 (12.5)	0.32
Psychologist	12 (15.2)	8 (34.8)	4 (7.1)	0.004
Post-COVID clinic	7 (8.9)	4 (17.4)	3 (5.4)	0.19
Rehabilitation	6 (7.6)	4 (17.4)	2 (3.6)	0.06
Neuropsychology	5 (6.3)	3 (13.0)	2 (3.6)	0.15
Post-intensive care unit follow-up clinic	4 (5.1)	4 (17.4)	0 (0.0)	0.01
Other	19 (24.1)	9 (39.1)	10 (17.9)	0.08
New medications prescribed by indication				
Heart problems	6 (7.6)	1 (4.4)	5 (8.9)	0.67
Lung problems	6 (7.6)	2 (8.7)	4 (7.1)	1.00
Seizures	5 (6.3)	3 (13.0)	2 (3.6)	0.15
Steroids	5 (6.3)	4 (17.4)	1 (1.8)	0.02
Anxiety	4 (5.1)	3 (13.0)	1 (1.8)	0.07
Depression	3 (3.8)	2 (8.7)	1 (1.8)	0.20
Pain	3 (3.4)	2 (8.7)	1 (1.8)	0.20
Antibiotics	3 (3.8)	2 (8.7)	1 (1.8)	0.20
Other psychiatric (mood, antipsychotics)	3 (3.8)	1 (4.4)	2 (3.6)	1.00
Postural orthostatic tachycardia syndrome	0 (0.0)	0 (0.0)	0 (0.0)	–
Drug withdrawal	0 (0.0)	0 (0.0)	0 (0.0)	–

### Post-hospital symptoms

Overall, two-thirds of patients had at least one symptom that was new (27.4%) or continued (56.3%) after hospital discharge (Table 2) (Supplementary Table S3).

**Table 2:** New or persistent symptoms at the time of survey completion post-hospital discharge by recovered status. Symptoms are grouped by organ system^a^.

Category/symptom, *n* (%)	Overall *N* = 79	Not recovered *N* = 23 (29.1%)	Recovered *N* = 56 (70.9%)	*P*-value*
Any symptom	53 (67.1)	16 (69.6)	37 (66.1)	1.000
Neurologic	45 (49.5)	20 (87.0)	17 (30.4)	<0.001
Headache	23 (25.4)	14 (60.9)	6 (10.8)	<0.001
Weakness	20 (25.3)	11 (47.8)	9 (16.1)	0.01
Balance problems	12 (15.2)	7 (30.4)	5 (8.9)	0.03
Loss of smell	11 (13.9)	7 (30.4)	4 (7.1)	0.01
Loss of taste	9 (11.4)	6 (26.1)	3 (5.4)	0.02
Tingling sensations	9 (11.4)	7 (30.4)	2 (3.6)	0.002
Shooting or burning pain	8 (10.1)	6 (26.1)	2 (3.6)	0.01
Dizziness (room is spinning)	6 (7.6)	5 (21.7)	1 (1.8)	0.01
Numbness	5 (6.4)	5 (21.8)	0 (0.0)	0.001
Vision problems	4 (5.1)	3 (13.1)	0 (0.0)	0.07
Seizures	4 (5.1)	1 (4.4)	3 (5.4)	1.00
Delirium or confusion	4 (5.1)	3 (13.1)	1 (1.8)	0.07
Physical	28 (30.8)	14 (60.9)	9 (16.1)	<0.001
Muscle aches or pain	17 (21.6)	13 (56.5)	4 (7.1)	<0.001
Joint pain	14 (17.7)	9 (38.1)	5 (8.9)	0.003
Swallowing/chewing problem	6 (7.6)	3 (13.1)	3 (5.4)	0.35
Cognitive	20 (22.0)	11 (47.8)	4 (7.1)	<0.001
Thinking or concentration problem	10 (12.7)	8 (34.8)	2 (3.6)	0.001
Trouble with remembering things	9 (11.4)	8 (34.7)	1 (1.8)	<0.001
Speaking/communicating problem	7 (8.9)	5 (21.8)	2 (3.6)	0.02
Emotional	25 (27.5)	13 (56.5)	7 (12.5)	
Anxious	19 (24.1)	12 (52.1)	7 (12.5)	<0.001
Sad or depressed	11 (14.0)	7 (30.4)	4 (7.1)	0.01
General	41 (45.1)	15 (65.2)	20 (35.7)	0.02
Fatigue or tiredness	31 (39.3)	14 (60.9)	17 (30.4)	0.02
Fever	17 (21.5)	8 (34.8)	9 (16.1)	0.08
Loss of appetite	15 (19.0)	7 (30.5)	8 (14.3)	0.12
Sleep problems	14 (17.7)	9 (39.1)	5 (8.9)	0.003
Throat pain	9 (11.4)	6 (26.1)	3 (5.4)	0.02
Swollen glands	3 (3.8)	3 (13.1)	0 (0.0)	0.02
Respiratory	27 (29.7)	9 (39.1)	13 (23.2)	0.17
Cough	18 (22.8)	9 (39.1)	9 (16.1)	0.04
Trouble breathing	15 (19.0)	8 (34.8)	7 (12.5)	0.03
Gastrointestinal	28 (30.8)	13 (56.5)	10 (17.9)	0.00
Abdominal pain	16 (20.3)	9 (39.1)	7 (12.5)	0.01
Diarrhea	15 (19.0)	8 (34.7)	7 (12.5)	0.03
Vomiting or nausea	13 (16.4)	8 (34.8)	5 (8.9)	0.01
Cardiovascular	21 (23.1)	10 (43.5)	5 (8.9)	0.01
Palpitations	10 (12.7)	7 (30.5)	3 (5.4)	0.01
Lightheadedness or fainting	8 (10.1)	6 (26.0)	2 (3.6)	0.01

^a^ From the Case Report Form: “For each of the symptoms listed below, please answer whether your child had any new or continuing symptoms after the hospitalization that included a COVID-19 or MIS-C diagnosis. You can type in other symptoms at the bottom of this section”.
• Yes, symptom occurred in the hospital and continued after discharge** • Yes, new symptom that started after hospital discharge** • No*** • Unsure***

* *p*-value for any symptom (new or persistent) vs. none.

** Combined for analysis.

*** Combined with No for analysis.

There were no group differences in the frequency of new/continued symptoms (66.1% vs. 69.6%, *p* = 1.000). By category, the frequency of new/continued symptoms was more common in the not recovered vs. recovered groups, except for the respiratory category. Headaches were the most frequent new symptom reported for children recovered (5.4%) with very few others reported. Among children not recovered, new symptoms most frequently reported were anxiousness or sleep problems (both 21.7%). The most common continued symptoms for children who were recovered were fatigue (28.6%), weakness (16.1%), cough (16.1%), fever (14.3%), and loss of appetite (14.3%), while for children not recovered, they were fatigue (47.8%), headache (43.5%), muscle aches (39.1%), and weakness (39.1%).

### Post-hospitalization healthcare utilization

Thirty-one (39.2%) children had at least one emergency department visit since hospital discharge, with a median of 2 (IQR 1, 3) visits (Table 3). There were no group differences in the occurrence of emergency department or hospital readmission.

Post-discharge care was most frequently provided by the child's primary care provider (73.4%), followed by cardiology (35.4%) and neurology (22.8%).

New medications were prescribed post-hospital discharge for heart and lung problems (both 7.6%) and seizures (6.3%). Of the children prescribed steroids (6.3%), more children were not recovered vs. recovered (17.4% vs. 1.8%, *p* = 0.02).

### Post-hospital child and family outcomes

Total HRQOL [87 (77, 95) vs. 77 (51, 83), *p* = 0.005] was better in children who were recovered vs. not recovered (Table 4). Subdomain scores were similarly better for the recovered group except for school scores (*n* = 23 ag ≥2 years), which were not different between groups.

**Table 4:** Univariate and multivariable logistic regressions associated with recovered status.

Variable	*N*	Univariate odds ratio^a^	95% confidence interval	*p*-value	Multivariable odds ratio	95% confidence interval	*p*-value
Any new or persistent symptom	79	0.85	0.30, 2.43	0.76			
Total family impact module score	74	1.02	0.99, 1.04	0.17			
Hospital length of stay	79	1.05	0.95, 1.16	0.33			
Sex	79	0.56	0.21, 1.50	0.25			
Age	78	0.93	0.85, 1.02	0.11			
Race	77	0.60	0.33, 1.08	0.09			
Ethnicity	66	1.24	0.23, 6.79	0.80			
Neurologic comorbidity	79	0.996	0.34, 2.86	1.00			
Non-neurologic comorbidity	77	1.62	0.54, 4.80	0.39			
Any comorbidity	79	2.10	0.78, 5.70	0.14			
Glasgow coma scale score, initial	67	0.96	0.76, 1.20	0.71			
Pediatric index of mortality	35	0.95	0.86, 1.05	0.30			
Pediatric logistic organ dysfunction score	35	0.96	0.87, 1.06	0.43			
Cerebrospinal fluid: white blood cell count	7	1.01	0.93, 1.10	0.80			
Sodium	71	1.04	0.91, 1.20	0.54			
Hemoglobin	68	1.03	0.81, 1.32	0.81			
C-reactive protein	47	0.92	0.86, 0.99	0.02	0.93	0.86, 0.99	0.03
Ferritin	37	0.9996	0.998, 1.00	0.60			
Procalcitonin	27	0.99	0.93, 1.05	0.64			
Fibrinogen^b^	31	0.995	0.99, 1.00	0.07			
Alanine transaminase	57	1.00	0.997, 1.00	0.68			
Aspartate transaminase	53	1.00	0.995, 1.01	0.71			
Prothrombin	31	1.23	0.88, 1.72	0.23			
Partial thromboplastin time	37	1.00	0.96, 1.05	0.95			
International normalized ratio	37	2.21	0.05, 90.08	0.68			
D-dimer	32	1.00	0.72, 1.40	0.99			
Steroids	79	0.39	0.14, 1.10	0.08			
Remdesivir	79	0.91	0.25, 3.31	0.89			
Intravenous immune globulin	79	0.44	0.14, 1.37	0.16			
Any treatment	79	0.44	0.16, 1.19	0.11			
Epoch	79	0.92	0.43, 2.00	0.84			
COVID vs. MIS-C	79	0.50	0.16, 1.52	0.22			
Any neuromanifestation	79	0.83	0.31, 2.19	0.70			
Severe neuromanifestation	79	0.996	0.35, 2.86	1.00			
White blood cells	68	1.00	0.9999, 1.00	0.64			
Lymphocytes	65	1.0005	0.9999, 1.001	0.05	1.001	1.0001, 1.002	0.03
Platelets	67	1.00	0.999, 1.000	0.88			
Impairment^c^	77	2.34	0.27, 21.22	0.43			
Intensive care unit vs. ward	79	1.98	0.68, 5.77	0.21			
Coronavirus impact scale score	73	0.88	0.78, 0.99	0.03	0.86	0.71, 1.04	0.12

^a^ Variables considered for inclusion if univariate *p* < 0.1.

^b^ Variable not included in multivariate due to missing >50%.

^c^ New impairment at hospital discharge was defined as any baseline to hospital discharge worsening in either the Pediatric Cerebral Performance Category (PCPC) or Functional Status Scale (FSS) score, or discharge PCPC > 1 or FSS > 6.MIS-C, multisystem inflammatory syndrome in children.

Global functioning as assessed by FSS was better in recovered vs. not recovered groups [6 (6, 6) vs. 6 (6, 8), *p* = 0.01], but PROMIS measures were not different (Supplementary Tables S5, S6).

Family functioning total score was not different between recovered groups [78 (65, 93) vs. 73 (60, 82), *p* = 0.13] (Supplementary Table S7).

### Sociocultural outcomes

Many families reported changes in their family's socioeconomics and mental health statuses (Supplementary Table S8). The sub-domains in which the largest numbers of families reported any change included routines (e.g., work, education, social life, hobbies, religious activities) (90.4%) and stress related to the pandemic (83.6%). For changes in routine, most families reported a severe status change with a change in 3 or more categories (32.9%). Parents with children who were not recovered had a worse CIS score than those with recovered children [8 (5, 13) vs. 6 (4, 9), *p* = 0.03]. They also reported changes in food access (47.6% vs. 21.1%, *p* = 0.01), accessing mental health treatment (47.6% vs. 19.2%, *p* = 0.002), and stress due to the pandemic (100.0% vs. 76.9%, *p* = 0.04).

### School and vaccination status

In the children >5 years of age (*n* = 50), 80.0% of children were back to exclusive in-person schooling and 14% were exclusively homeschooled (Supplementary Table S9). There was no difference in vaccination status between groups at the time of the survey.

### Multivariable logistic regression analyses for recovered status and HRQOL

In a multivariable logistic regression, lower CRP [adjusted odds ratio (aOR) 0.90, 95% confidence interval 0.82, 0.99] and higher lymphocyte count (aOR 1.001, 95% CI 2.37–5.15) on hospital admission were associated with recovered status (Table 4). These results were unchanged when acute SARS-CoV-2 vs. MIS-C was forced into the model (data not shown).

A total of 12 out of 79 (15.2%) children had poor HRQOL at follow-up. In a multivariable logistic regression, worse family function scores (0.94, 0.91, 0.98) and neurologic comorbidity (10.57, 1.71, 65.16) were associated with poor HRQOL (Supplementary Table S10).

## Discussion

In this multicenter survey of families with children previously hospitalized with a SARS-CoV-2-related condition, we found the following: (1) Nearly a third of children were defined by parents as being not recovered; (2) New or continued symptoms post-discharge occurred in two-thirds of children overall, with symptoms most prevalent in children not recovered; (3) Return emergency and hospital admissions were common with follow-up care mostly provided by primary care clinicians; (4) Children not recovered had worse overall function and HRQOL; (5) Families with children not recovered had more pandemic-related social stress adversities; and (6) Increased inflammation and decreased lymphocyte counts at hospital admission were associated with not recovered status.

Nearly 30% of parents reported their child as being not recovered at a median of 1.5 years following hospitalization. Published studies of COVID-19 Syndrome in children have reported prevalence rates of 1%–66%, with higher risk in children associated with age <5 years, adolescent age, and increased severity of illness (6, 18, 30–43). Our study's finding that two-thirds of children were experiencing either new or continued symptoms is similar to previously published surveys (6). Prior work evaluating Post-COVID-19 Syndrome used various designs (e.g., single center (39), community (34), and national (30, 38, 41, 42), definitions, data sources (e.g., proxy or child report surveys or in-person assessments (32, 36, 37, 42), electronic health record (31), and insurance registries (18), ages (e.g., inclusive pediatric age range (37) vs. adolescents( 40, 42), specific SARS-CoV-2-related condition (33, 35–37), level of care, and time points, contributing to the wide-ranging prevalence estimates.

Two-thirds of children had any reported new or persistent symptoms in our study at a median of 1 year post-discharge compared to the Overcoming COVID follow-up study, where the prevalence of any symptoms was 22.7% for COVID-19% and 20% for MIS-C patients at 2–4 months, and in a recent systematic review of hospitalized and non-hospitalized children where symptom prevalence was 16.2% (95% confidence interval 8.5%–28.6%) (37, 43). Symptoms in our study were mostly persistent since discharge, with children who were not recovered having vastly more symptoms. Strikingly, 87% of the not recovered group had a neurologic symptom, especially headache (60.9%) and weakness (47.8%). Other common symptoms in the not recovered group were fatigue (60.9%), muscle aches or pains (56.5%), and anxiousness (52.1%). These domains are similar to the Overcoming COVID follow-up study which reported that fatigue/weakness (14.3% COVID-19 and 11.3% MIS-C) was the most common symptom (37). Further, we found problems with thinking or concentration, trouble remembering things, and sleep problems were common in children not recovered. Finally, 30.4% of children reported as recovered had fatigue and 16.1% each had weakness, fever, or cough, similar to surveys of healthy control populations (40, 42). Emotional and mental health impairments related to the pandemic are likely reflected in our findings where more than half of parents reported their child being anxious and 30% as being sad in the not recovered group (44, 45). The latter psychosocial symptoms may help explain why the prevalence of fatigue in recovered children in our study is much higher than previously reported in healthy populations (46, 47).

Hospital-based treatment guidelines for pediatric COVID-19 patients include steroids, which reduced hospital mortality in critically ill adults with COVID-19, and both IVIG and steroids for MIS-C (48–51). In one study of MIS-C patients, steroids were not associated with later neuropsychological outcomes (36). Also, steroids, IVIG, and remdesivir were not associated with recovered status or HRQOL in children with COVID-19 (52).

Healthcare utilization post-discharge was substantial. More than half of the children in the not recovered group and a third of those in the recovered group had at least one emergency department visit, with a median of 2 visits overall. Nearly a quarter of patients had at least one hospital readmission, with a median of 4 vs. 1.5 admissions in the not recovered and recovered groups, respectively, potentially contributing to a child's recovery status. A study with similar cohort characteristics found that 11% of acute COVID and 8% of MIS-C patients had hospital readmissions 2–4 months post-discharge (37). Furthermore, children reported as not recovered were more likely to receive primary care, neurology, pulmonology, psychology, and post-ICU follow-up clinic services, demonstrating an urgent need for educational efforts regarding screening and care for the child with increased risk of Post-COVID-19 Syndrome (53). New medications prescribed to treat SARS-CoV-2-related conditions post-discharge were uncommon. These results may reflect the lack of evidence-based treatments for Post-COVID-19 syndrome in children; the Centers for Disease Control and Prevention in the U.S. recommends a patient-centered, multidisciplinary approach to the care of those with Post-COVID-19 Syndrome (2, 54). Also, COVID-19 vaccination is associated with decreased risk of Post-COVID-19 Syndrome (55–57) but patients in this study were not eligible for vaccination prior to initial hospitalization.

Gross measures of child function were mostly similar by recovered status; detailed neuropsychological outcomes testing research is limited, but two small studies found impairments in children with MIS-C (35, 36). Our study found that HRQOL impairment persists well past 4 months given our longer follow-up period especially for children not recovered. These findings are similar to a study in MIS-C patients from 7 PICUs in the Netherlands where physical and school functioning HRQOL subdomains at a median of 4 months post-discharge were worse in comparison to population norms (35). Finally, access to care and social determinants of health affect health outcomes (58, 59). Worse CIS scores were associated with not recovered status and poor HRQOL in this study.

In our multivariate analyses, we sought to identify factors associated with non-recovered status and poor HRQOL. A leading hypothesis of Post-COVID-19 Syndrome etiology is persistent virus and inflammation (2, 60). Children in the not recovered group had increased inflammation at hospital admission as indicated by blood CRP levels and decreased lymphocytes on complete blood counts. These laboratory findings were associated with MIS-C vs. COVID-19 diagnosis in two prior studies, whereas comparison of laboratory signatures in our cohort by condition was limited by sample size and imbalance of MIS-C vs. acute SARS-CoV-2 (61–63). In a multicenter study of children with severe sepsis, persistent lymphopenia was associated with organ failure and death and higher maximal CRP levels (64). We also found that family dysfunction and a child's preexisting neurologic comorbidity status were associated with worse child HRQOL post-discharge. Previous studies describe that family function mitigates child outcomes after pediatric traumatic brain injury (65, 66). Also, our results show that children with neurologic comorbidity were more likely to have neurologic manifestations during hospitalization, with long-term outcomes data previously lacking (21, 67). Notably, SARS-CoV-2 infection is now so common that linking a remote infection to subsequent common neurologic symptoms such as headache, seizures, or fatigue in the outpatient setting is challenging as there may be no causal relationship. The lack of identified mechanisms for SARS-CoV-2-related neurologic injury exacerbates this issue.

The clinical implications of our findings are vast and should be validated longitudinally in rigorous prospective patient cohorts using contemporary Post-COVID-19 syndrome definitions (1, 7, 16, 68). Further, laboratory values such as CRP could be tested as predictors of Post-COVID-19 Syndrome. Our work implies that coordinated, longitudinal child and family support may be needed to optimally prevent and treat Post-COVID-19 Syndrome in children (69).

### Limitations

The generalization of findings from this work is limited by the fact that participating centers are only from the U.S. and the sample size is relatively small and at risk of response bias. General pandemic effects and other primary diagnoses (e.g., trauma) upon index hospitalization may have affected outcomes (20, 70). Limitations include nonresponse bias and parental recall bias of symptoms and recovered status. This research study lacks a control group and does not include a detailed neuropsychological assessment.

## Conclusions

In this multicenter parent-report survey of children previously hospitalized with acute SARS-CoV-2 and MIS-C, one-third of children were reported as not recovered. Not recovered status was associated with multidomain symptomatology, increased healthcare utilization, and worse HRQOL. Longitudinal, coordinated follow-up is required to assess Post-COVID-19 Syndrome for appropriate referral and care to support recovery.

## Data Availability

The datasets presented in this article are not readily available because data use agreements with sites disallowed data sharing. Requests to access the datasets should be directed to finkel@ccm.upmc.edu.

## GCS-NeuroCOVID-Pediatrics Investigators

Research Coordinators: Bianca Pate, BA, Pamela Rubin, RN (UPMC Children's Hospital of Pittsburgh); Lisa Steele RN, BSN, CCRN (Nationwide Children's Hospital); Jacqueline Lee-Eng BSc, Mikaela Gatterman, R. EEG T (Seattle Children's Hospital); Ben Orwoll, MD (Oregon Health & Science University); Paige Selenski, Lensy Arce Porior (University of Wisconsin); Ronke Awojoodu MPH, BSN, Colleen Mennie RN, BSN (John Hopkins University); Ria Pal, MD (Stanford University); Geetika Chahal, MBBS (Phoenix Children's Hospital); Kristy Wei, MPH (University of Chicago); Aubrie Waters, BS and Caitlin Stoll, DO (MUSC Shawn Jenkins Children's Hospital). University of Pittsburgh Data Coordinating Center [Department of Critical Care Medicine's Clinical Research, Investigation, and Systems Modeling of Acute Illness (CRISMA) center and UPMC Children's Hospital of Pittsburgh's Division of Pediatric Critical Care Medicine]: Research program staff: Nicole Toney, MPH; Ali Smith Scott, BA. Data coordination: Dan Ricketts, MET; Edvin Music, MSIS, MBA; Jonathan Holton, MSIS. GCS-NeuroCOVID Consortium Steering Committee: Sherry H.-Y. Chou, MD, MSc (Northwestern University—Feinberg School of Medicine, USA); Raimund Helbok, MD (Medical University of Innsbruck, Innsbruck, Austria); Paul Vespa MD (University of California, Los Angeles, USA); Daiwai Olson RN PhD (University of Texas Southwestern, USA); Claude Hemphill MD (University of California, San Francisco, USA); Chethan P Venkatasubba Rao MD (Baylor College of Medicine, USA); Nerissa Ko MD MS (University of California, San Francisco, USA); Jose I. Suarez, MD (The Johns Hopkins University School of Medicine, USA); Shraddha Mainali, MD (Virginia Commonwealth University, USA); Molly McNett, PhD (The Ohio State University, USA).
