# Corrigendum: Post-discharge outcomes of hospitalized children diagnosed with acute SARS-CoV-2 or MIS-C

**DOI:** 10.3389/fped.2024.1440609

**Published:** 2024-07-01

**Authors:** Ericka L. Fink, Alicia M. Alcamo, Marlina Lovett, Mary Hartman, Cydni Williams, Angela Garcia, Lindsey Rasmussen, Ria Pal, Kurt Drury, Elizabeth MackDiaz, Peter A. Ferrazzano, Leslie Dervan, Brian Appavu, Kellie Snooks, Casey Stulce, Pamela Rubin, Bianca Pate, Nicole Toney, Courtney L. Robertson, Mark S. Wainwright, Juan D. Roa, Michelle E. Schober, Beth S. Slomine

**Affiliations:** ^1^ Department of Critical Care Medicine, UPMC Children’s Hospital of Pittsburgh, Pittsburgh, PA, United States; ^2^ Safar Center for Resuscitation Research, University of Pittsburgh Medical Center, Pittsburgh, PA, United States; ^3^ Department of Anesthesiology and Critical Care Medicine, Children’s Hospital of Philadelphia, University of Pennsylvania, Philadelphia, PA, United States; ^4^ Division of Critical Care Medicine, Department of Pediatrics, Nationwide Children’s Hospital, The Ohio State University College of Medicine, Columbus, OH, United States; ^5^ Division of Pediatric Critical Care Medicine, Seattle Children’s Hospital, University of Washington, Seattle, WA, United States; ^6^ Department of Pediatrics, Pediatric Critical Care and Neurotrauma Recovery Program, Oregon Health & Science University, Portland, OR, United States; ^7^ Division of Pediatric Physical Medicine and Rehabilitation, UPMC Children’s Hospital of Pittsburgh, Pittsburgh, PA, United States; ^8^ Division of Pediatric Critical Care Medicine, Lucile Packard Children’s Hospital, Stanford University, Palo Alto, CA, United States; ^9^ Department of Neurology, Lucile Packard Children’s Hospital, Stanford University, Palo Alto, CA, United States; ^10^ Division of Pediatrics, Comer Children’s Hospital, University of Chicago, Chicago, IL, United States; ^11^ Division of Pediatric Critical Care Medicine, MUSC Shawn Jenkins Children’s Hospital, Charleston, SC, United States; ^12^ Department of Pediatrics, University of Wisconsin, Madison, WI, United States; ^13^ Division of Pediatric Critical Care Medicine, Department of Pediatrics, University of Washington School of Medicine, Seattle, WA, United States; ^14^ Division of Neurology, Barrow Neurological Institute at Phoenix Children’s Hospital, College of Medicine, University of Arizona, Phoenix, AZ, United States; ^15^ Department of Pediatrics, Medical College of Wisconsin, Milwaukee, WI, United States; ^16^ Department of Pediatrics, University of Chicago, Chicago, IL, United States; ^17^ Departments of Anesthesiology and Critical Care Medicine, and Pediatrics, Johns Hopkins Children’s Center, Baltimore, MD, United States; ^18^ Division of Pediatric Neurology, Seattle Children’s Hospital, University of Washington, Seattle, WA, United States; ^19^ Department of Pediatrics, Universidad Nacional de Colombia and Fundación Universitaria de Ciencias de la Salud, Bogotá, Colombia; ^20^ Division of Critical Care, Department of Pediatrics, University of Utah, Salt Lake City, UT, United States; ^21^ Department of Psychiatry and Behavioral Sciences, Kennedy Krieger Institute, Johns Hopkins University School of Medicine, Baltimore, MD, United States

**Keywords:** pediatrics, SARS-CoV-2, child development, patient outcome assessment, post-acute COVID-19 syndrome

A Corrigendum on Post-discharge outcomes of hospitalized children diagnosed with acute SARS-CoV-2 or MIS-C By Fink EL, Alcamo AM, Lovett M, Hartman M, Williams C, Garcia A, Rasmussen L, Pal R, Drury K, MackDiaz E, Ferrazzano PA, Dervan L, Appavu B, Snooks K, Stulce C, Rubin P, Pate B, Toney N, Robertson CL, Wainwright MS, Roa JD, Schober ME and Slomine BS (2024). Front. Pediatr. **12**:1340385. doi: 10.3389/fped.2024.1340385

In the published article, an author name was incorrectly written as Brain Appavu. The correct spelling is Brian Appavu.

The authors apologize for this error and state that this does not change the scientific conclusions of the article in any way. The original article has been updated.

