# Supplementary material for: Post-discharge outcomes of hospitalized children diagnosed with acute SARS-CoV-2 or MIS-C
Source: Front Pediatr. 2024 Feb 12;12:1340385. doi: 10.3389/fped.2024.1340385 (PMC10895015; doi:10.3389/fped.2024.1340385)
Supplement: Supplementary file 1 [file Table1.docx]

**Supplemental Table 1**. Characteristics of Tier 1 and Tier 2 cohorts of the GCS-NeuroCOVID Pediatrics study.

| Variables | Tier 1  N=3,568 | Tier 2  N=79 |
| --- | --- | --- |
| **Epoch** | N=3,363 |  |
| January 2020-June 2020 | 600 (17.8) | 6 (7.6) |
| July 2020-December 2020 | 1629 (48.4) | 31 (39.2) |
| January 2021-July 2021 | 1134 (33.7) | 42 (53.2) |
| **Age, y** | 8 (1, 14)  N=3560 | 6.5 (2.0, 13.0) |
| **Sex** | N=3565 |  |
| Female | 1627 (45.6) | 30 (38.0) |
| Male | 1938 (54.4) | 49 (62.0) |
| **Race** | N=3516 |  |
| Asian | 275 (7.8) | 4 (5.2) |
| Black or African American | 820 (23.3) | 17 (22.1) |
| White | 1858 (52.8) | 53 (68.8) |
| Other | 563 (16.0) | 3 (3.9) |
| **Hispanic Ethnicity** | 1024 (31.1)  N=3295 | 8 (12.1) |
| **Acute SARS-CoV-2 (vs. MIS-C)** | 2604 (81.6) | 53 (75.7) |
| **Pre-existing condition** |  |  |
| Neurologic | 686 (19.3)  N=3553 | 24 (30.4) |
| Non-neurologic | 1360 (38.2)  N=3563 | 28 (36.4) |
| **Highest level of care** |  |  |
| Intensive care unit | 1635 (45.8) | 29 (36.7) |
| Ward | 1933 (54.2) | 50 (63.3) |
| **Hospital length of stay, d** | 4 (2, 8)  N=3558 | 3 (2, 8) |
| **ICU length of stay, d** | 1 (2, 6)  N=1115 | 1. (2, 5) |
| **Hospital mortality** | 40 (1.2) | 0 (0) |
| **Home disposition post-hospital discharge** | 3362 (94.4) | 77 (97.5) |
| **Any neurologic manifestation** | 1385 (38.8) | 42 (53.2) |
| **Impairment at hospital discharge** | 605 (17.0) | 7 (9.1) |

**Supplemental Table 2**. Survey outcome measures, domains, estimated time to complete, and respondents.

| **Measure** | **Domain** | **Time (min)** | **Respondent** |
| --- | --- | --- | --- |
| **Functional Status Scale** | Global function | < 5 | Parent/proxy |
| **Pediatric Cerebral Performance Category** | Neurologic function | < 5 | Parent/proxy |
| **PedsQL Infant/Child Core** | HRQOL | 5 | Parent/proxy |
| **Peds QL Family Impact Module** | Family impact | 15 | Parent/proxy |
| **Symptoms, Utilization, Meds, Participation, Unmet needs, School, Vaccination** | Healthcare utilization  PROs | <5 | Parent/proxy |
| **PROMIS Parent Proxy Global Health** | Global function | <5 | Parent/proxy |
| **Coronavirus Impact Scale** | Family impact specific to pandemic, SES | <5 | Parent/proxy |

**Supplemental Table 3.** New, persistent, or either new or persistent symptoms at the time of survey completion post-hospital discharge. Symptoms are grouped by organ system.

|  | Overall  N=79 | | | Not recovered  N=23 (29.1%) | | | Recovered  N=56 (70.9%) | | |
| --- | --- | --- | --- | --- | --- | --- | --- | --- | --- |
| **Category/Symptom** | New post-HD | Continued post-HD | **New / Continued** | New post-HD | Continued post-HD | **New / Continued** | New post-HD | Continued post-HD | **New / Continued** |
| **Any symptom** | 23 (27.4) | 49 (56.3) | 53 (67.1) | 11 (64.7) | 19 (90.5) | 16 (69.6) | 9 (16.4) | 27 (50.0) | 37 (66.1) |
| **Neurologic** |  |  | 45 (49.5) |  |  | 20 (87.0) |  |  | 17 (30.4) |
| Headache | 7 (8.9) | 13 (16.5) | 23 (25.4) | 4 (17.4) | 10 (43.5) | 14 (60.9) | 3 (5.4) | 3 (5.4) | 6 (10.8) |
| Weakness | 2 (2.5) | 18 (22.8) | 20 (25.3) | 2 (8.7) | 9 (39.1) | 11 (47.8) | 0 (0.0) | 9 (16.1) | 9 (16.1) |
| Balance problems | 5 (6.3) | 7 (8.9) | 12 (15.2) | 4 (17.4) | 3 (13.0) | 7 (30.4) | 1 (1.8) | 4 (7.1) | 5 (8.9) |
| Loss of smell | 0 (0.0) | 11 (13.9) | 11 (13.9) | 0 (0.0) | 7 (30.4) | 7 (30.4) | 0 (0.0) | 4 (7.1) | 4 (7.1) |
| Loss of taste | 0 (0.0) | 9 (11.4) | 9 (11.4) | 0 (0.0) | 6 (26.1) | 6 (26.1) | 0 (0.0) | 3 (5.4) | 3 (5.4) |
| Tingling sensations | 3 (3.8) | 6 (7.6) | 9 (11.4) | 3 (13.0) | 4 (17.4) | 7 (30.4) | 0 (0.0) | 2 (3.6) | 2 (3.6) |
| Shooting or burning pain | 2 (2.5) | 6 (7.6) | 8 (10.1) | 2 (8.7) | 4 (17.4) | 6 (26.1) | 0 (0.0) | 2 (3.6) | 2 (3.6) |
| Dizziness (room is spinning) | 3 (3.8) | 3 (3.8) | 6 (7.6) | 3 (13.0) | 2 (8.7) | 5 (21.7) | 0 (0.0) | 1 (1.8) | 1 (1.8) |
| Numbness | 1 (1.3) | 4 (5.1) | 5 (6.4) | 1 (4.4) | 4 (17.4) | 5 (21.8) | 0 (0.0) | 0 (0.0) | 0 (0.0) |
| Vision problems | 3 (3.8) | 1 (1.3) | 4 (5.1) | 2 (8.7) | 1 (4.4) | 3 (13.1) | 1 (1.8) | 0 (0.0) | 0 (0.0) |
| Seizures | 1 (1.3) | 3 (3.8) | 4 (5.1) | 0 (0.0) | 1 (4.4) | 1 (4.4) | 1 (1.8) | 2 (3.6) | 3 (5.4) |
| Delirium or confusion | 1 (1.3) | 3 (3.8) | 4 (5.1) | 1 (4.4) | 2 (8.7) | 3 (13.1) | 0 (0.0) | 1 (1.8) | 1 (1.8) |
| **Physical** |  |  | 28 (30.8) |  |  | 14 (60.9) |  |  | 9 (16.1) |
| Muscle aches or pain | 4 (5.1) | 13 (16.5) | 17 (21.6) | 4 (17.4) | 9 (39.1) | 13 (56.5) | 0 (0.0) | 4 (7.1) | 4 (7.1) |
| Joint pain | 5 (6.3) | 9 (11.4) | 14 (17.7) | 4 (17.4) | 5 (21.7) | 9 (38.1) | 1 (1.8) | 4 (7.1) | 5 (8.9) |
| Swallowing / chewing problem | 1 (1.3) | 5 (6.3) | 6 (7.6) | 1 (4.4) | 2 (8.7) | 3 (13.1) | 0 (0.0) | 3 (5.4) | 3 (5.4) |
| **Cognitive** |  |  | 20 (22.0) |  |  | 11 (47.8) |  |  | 4 (7.1) |
| Thinking or concentration problem | 4 (5.1) | 6 (7.6) | 10 (12.7) | 4 (17.4) | 4 (17.4) | 8 (34.8) | 0 (0.0) | 2 (3.6) | 2 (3.6) |
| Trouble with remembering things | 3 (3.8) | 6 (7.6) | 9 (11.4) | 3 (13.0) | 5 (21.7) | 8 (34.7) | 0 (0.0) | 1 (1.8) | 1 (1.8) |
| Speaking / communicating problem | 4 (5.1) | 3 (3.8) | 7 (8.9) | 4 (17.4) | 1 (4.4) | 5 (21.8) | 0 (0.0) | 2 (3.6) | 2 (3.6) |
| **Emotional** |  |  | 25 (27.5) |  |  | 13 (56.5) |  |  | 7 (12.5) |
| Anxious | 7 (8.9) | 12 (15.2) | 19 (24.1) | 5 (21.7) | 7 (30.4) | 12 (52.1) | 2 (3.6) | 5 (8.9) | 7 (12.5) |
| Sad or depressed | 4 (5.1) | 7 (8.9) | 11 (14.0) | 4 (17.4) | 3 (13.0) | 7 (30.4) | 0 (0.0) | 4 (7.1) | 4 (7.1) |
| **General** |  |  | 41 (45.1) |  |  | 15 (65.2) |  |  | 20 (35.7) |
| Fatigue or tiredness | 4 (5.1) | 27 (34.2) | 31 (39.3) | 3 (13.0) | 11 (47.8) | 14 (60.9) | 1 (1.8) | 16 (28.6) | 17 (30.4) |
| Fever | 3 (3.8) | 14 (17.7) | 17 (21.5) | 2 (8.7) | 6 (26.1) | 8 (34.8) | 1 (1.8) | 8 (14.3) | 9 (16.1) |
| Loss of appetite | 1 (1.3) | 14 (17.7) | 15 (19.0) | 1 (4.4) | 6 (26.1) | 7 (30.5) | 0 (0.0) | 8 (14.3) | 8 (14.3) |
| Sleep problems | 5 (6.3) | 9 (11.4) | 14 (17.7) | 5 (21.7) | 4 (17.4) | 9 (39.1) | 0 (0.0) | 5 (8.9) | 5 (8.9) |
| Throat Pain | 5 (6.3) | 4 (5.1) | 9 (11.4) | 4 (17.4) | 2 (8.7) | 6 (26.1) | 1 (1.8) | 2 (3.6) | 3 (5.4) |
| Swollen glands | 2 (2.5) | 1 (1.3) | 3 (3.8) | 2 (8.7) | 1 (4.4) | 3 (13.1) | 0 (0.0) | 0 (0.0) | 0 (0.0) |
| **Respiratory** |  |  | 27 (29.7) |  |  | 9 (39.1) |  |  | 13 (23.2) |
| Cough | 2 (2.5) | 16 (20.3) | 18 (22.8) | 2 (8.7) | 7 (30.4) | 9 (39.1) | 0 (0.0) | 9 (16.1) | 9 (16.1) |
| Trouble breathing | 2 (2.5) | 13 (16.5) | 15 (19.0) | 1 (4.4) | 7 (30.4) | 8 (34.8) | 1 (1.8) | 6 (10.7) | 7 (12.5) |
| **Gastrointestinal** |  |  | 28 (30.8) |  |  | 13 (56.5) |  |  | 10 (17.9) |
| Abdominal Pain | 3 (3.8) | 13 (16.5) | 16 (20.3) | 3 (13.0) | 6 (26.1) | 9 (39.1) | 0 (0.0) | 7 (12.5) | 7 (12.5) |
| Diarrhea | 3 (3.8) | 12 (15.2) | 15 (19.0) | 3 (13.0) | 5 (21.7) | 8 (34.7) | 0 (0.0) | 7 (12.5) | 7 (12.5) |
| Vomiting or nausea | 2 (2.5) | 11 (13.9) | 13 (16.4) | 2 (8.7) | 6 (26.1) | 8 (34.8) | 0 (0.0) | 5 (8.9) | 5 (8.9) |
| **Cardiovascular** |  |  | 21 (23.1) |  |  | 10 (43.5) |  |  | 5 (8.9) |
| Palpitations | 1 (1.3) | 9 (11.4) | 10 (12.7) | 1 (4.4) | 6 (26.1) | 7 (30.5) | 0 (0.0) | 3 (5.4) | 3 (5.4) |
| Lightheadedness or fainting | 3 (3.8) | 5 (6.3) | 8 (10.1) | 3 (13.0) | 3 (13.0) | 6 (26.0) | 0 (0.0) | 2 (3.6) | 2 (3.6) |

HD, hospital discharge

**Supplemental Table 4**. Pediatric health-related quality of life and subdomains overall and by fully recovered status.

| Overall and individual domains | Overall  N=76 | Not recovered  N=21 (27.6%) | Recovered  N=55 (72.4%) | P-value |
| --- | --- | --- | --- | --- |
| **Total** | 85 (73, 94) | 77 (51, 83) | 87 (77, 95) | 0.01 |
| **Physical** | 91 (75, 100) | 83 (53, 90) | 94 (84, 100) | 0.004 |
| **Emotional** | 88 (70, 100) | 70 (50, 94) | 90 (70, 100) | 0.01 |
| **Social** | 95 (80, 100) | 90 (60, 100) | 100 (85, 100) | 0.02 |
| **Cognitive** | 92 (71, 100) | 67 (46, 92) | 96 (83, 100) | 0.002 |
| **Psychosocial** | 83 (71, 94) | 73 (50, 82) | 89 (75, 98) | 0.002 |
| **School** ^1^ | 85 (53, 100), n=23 | 65 (55, 100), n=8 | 90 (50, 100), n=15 | 0.22 |

^1^ eligible if >= age 2 years

**Supplemental Table 5**. Functional Status Scale outcomes at the time of survey completion.

| Total and domains | Overall  N=78 | Not recovered  N=23 (29.5%) | Recovered  N=55 (70.5%) | p-value |
| --- | --- | --- | --- | --- |
| Total score | 6 (6, 7) | 6 (6, 8) | 6 (6, 6) | 0.01 |
| Mental |  |  |  | 0.05 |
| 1 | 69 (88.5) | 18 (78.3) | 51 (92.7) |  |
| 2 | 7 (9.0) | 3 (13.0) | 4 (7.3) |  |
| 3 | 2 (2.6) | 2 (8.7) | 0 (0.0) |  |
| Sensory |  |  |  | 1.00 |
| 1 | 73 (93.6) | 22 (95.7) | 51 (92.7) |  |
| 2 | 4 (5.1) | 1 (4.4) | 3 (5.5) |  |
| 5 | 1 (1.3) | 0 (0.0) | 1 (1.8) |  |
| Communication |  |  |  | 0.51 |
| 1 | 69 (88.5) | 19 (82.6) | 50 (90.9) |  |
| 2 | 3 (3.9) | 2 (8.7) | 1 (1.8) |  |
| 3 | 2 (2.6) | 1 (4.4) | 1 (1.8) |  |
| 4 | 1 (1.3) | 1 (4.4) | 1 (1.8) |  |
| 5 | 3 (3.9) | 0 (0.0) | 2 (3.6) |  |
| Motor |  |  |  | 0.49 |
| 1 | 72 (92.3) | 21 (91.3) | 51 (92.7) |  |
| 3 | 1 (1.3) | 1 (4.4) | 0 (0.0) |  |
| 4 | 2 (2.6) | 0 (0.0) | 2 (3.6) |  |
| 5 | 3 (3.9) | 1 (4.4) | 2 (3.6) |  |
| Feeding |  |  |  | 1.00 |
| 1 | 72 (92.3) | 22 (95.7) | 50 (90.9) |  |
| 2 | 1 (1.3) | 0 (0.0) | 1 (1.8) |  |
| 3 | 5 (6.4) | 1 (4.4) | 4 (7.3) |  |
| Respiratory |  |  |  | 0.84 |
| 1 | 72 (92.3) | 21 (91.3) | 51 (92.7) |  |
| 2 | 3 (3.9) | 1 (4.4) | 2 (3.6) |  |
| 3 | 1 (1.3) | 0 (0.0) | 1 (1.8) |  |
| 4 | 2 (2.6) | 1 (4.4) | 1 (1.8) |  |

**Supplemental Table 6**. Global child outcomes.

| Outcome | Overall  N=see subdomain | Not recovered  N=see subdomain | Recovered  N=see subdomain | P-value |
| --- | --- | --- | --- | --- |
| **PROMIS proxy total, n=46 (5-17 years of age)** ^4^ | 28 (24, 30) | 27.5 (26, 29), n=18 | 29 (24, 30), n=28 | 0.64 |
| General child health | 4 (3, 4)^1^ | 3 (3, 4) | 4 (3, 5) | 0.31 |
| General child quality of life | 4 (3, 5) ^1^ | 4 (3, 4) | 5 (3, 5) | 0.06 |
| Physical health | 4 (3, 5) ^1^ | 4 (3, 4) | 4 (3, 5) | 0.57 |
| Mental health (mood, ability to think) | 4 (3, 5) ^1^ | 3 (3, 4) | 4 (3, 5) | 0.10 |
| Fun with friends | 4 (3, 5) ^2^ | 4 (3, 5) | 4 (3, 5) | 0.54 |
| Child feel you listen to his/her ideas | 5 (4, 5) ^2^ | 5 (4, 5) | 5 (4, 5) | 0.90 |
| Child tires easily | 2 (1, 3) ^2^ | 2.5 (1, 4) | 1 (1, 3) | 0.06 |
| Trouble sleeping when he/she had pain | 1 (1, 3) ^3^ | 2 (1, 4) | 1 (1, 3) | 0.10 |
| Global total (10-30) | 24 (20, 27) | 24 (19, 25) | 24 (20, 27) | 0.33 |
| **PROMIS Young child, n=27 (1-4 years of age)** |  |  |  |  |
| General health | 4 (4, 5) ^1^ | 4.5 (4, 5), n=2 | 4 (4, 5), n=25 | 0.52 |
| General quality of life | 5 (4, 5) ^1^ | 4.5 (4, 5) | 5 (4, 5) | 0.92 |
| Physical | 4 (4, 5) ^1^ | 4.5 (4, 5) | 4 (4, 5) | 0.92 |
| Mental | 5 (4, 5) ^1^ | 5 (5, 5) | 5 (4, 5) | 0.25 |
| Mood | 4 (4, 5) ^1^ | 4.5 (4, 5) | 4 (4, 5) | 0.68 |
| Social skills | 4 (3, 5) ^1^ | 4.5 (4, 5) | 4 (3, 5) | 0.59 |
| Ability to think | 5 (4, 5) ^1^ | 5 (5, 5) | 5 (4, 5) | 0.25 |
| Meeting developmental milestones | 4 (3, 5) ^1^ | 4.5 (4, 5) | 4 (3, 5) | 0.56 |

^1^ Likert scale: 5=Excellent, 4=Very Good, 3=Good, 2=Fair, 1=Poor

^2^ Likert scale: 5=Always, 4=Often, 3=Sometimes, 2=Rarely, 1=Never

^3^ Likert scale: 5=Never, 4=Almost never, 3=Sometimes, 2=Often, 1=Almost always

^4^ The PROMIS question “Feel really sad” was omitted as an error in the study case report form

**Supplemental Table 7**. PedsQL Family Impact Module results.

| Family Impact Module domains | Overall  N=79 | Not recovered  N=23 (29.1%) | Recovered  N=56 (71.9%) | P-value |
| --- | --- | --- | --- | --- |
| **Physical** | 71 (50, 88) | 67 (50, 79) | 71 (54, 92) | 0.15 |
| **Emotional** | 75 (55, 90) | 70 (45, 85) | 75 (60, 90) | 0.14 |
| **Social** | 88 (63, 100) | 75 (63, 94) | 88 (69, 100) | 0.29 |
| **Cognitive** | 75 (60, 100) | 80 (70, 90) | 75 (60, 100) | 0.80 |
| **Communication** | 83 (58, 100) | 75 (42, 92) | 83 (67, 100) | 0.04 |
| **Worry** | 75 (50, 90) | 60 (40, 85) | 80 (55, 95) | 0.07 |
| **Daily activities** | 75 (50, 100) | 67 (50, 83) | 83 (58, 100) | 0.07 |
| **Family relationships** | 75 (65, 100) | 75 (55, 90) | 85 (65, 100) | 0.10 |
| **Parent health-related quality of life summary score** | 76 (62, 88) | 72 (61, 81) | 77 (63, 90) | 0.24 |
| **Family function summary score** | 78 (60, 100) | 70 (57, 85) | 84 (65, 100) | 0.06 |
| **Total score** | 75 (60, 89), n=74 | 73 (60, 82) | 78 (65, 93) | 0.13 |

Scoring:

Physical (n=6 questions), emotional (n=5), social (n=4), cognitive (n=5), communication (n=3), worry (n=5), daily activities (n=3), family relationships (n=5): section scores to be summed and divided by number of items answered to indicate average score 0-100

Parent health-related quality of life summary score: Compute the sum of the scores divided by the number of items answered in the Physical, Emotional, Social, and Cognitive Functioning scales (20 items); if <50% of items are entered, then cannot be scored

Family function summary score: Take the sum of the scores divided by the number of items answered in the Daily Activities and Family Relationships scales (8 items); if <50% of items are entered, then cannot be scored

Total Score: taking the sum of scores for all 36 items in the FIM measure divided by the number of items answered; if <50% of items are entered, the total score cannot be calculated

**Supplemental Table 8**. Coronavirus impact scale.

| Response to “How much has pandemic changed your life” | Overall  N=73 | Not recovered  N=21 (28.8%) | Recovered  N=52 (71.2%) | P-value |
| --- | --- | --- | --- | --- |
| **Overall Coronavirus Impact Scale score** | 6 (4, 10) | 8 (5, 13) | 6 (4, 9) | 0.03 |
| **Routines (e.g. work, education, social life, hobbies, religious activities)** |  |  |  | 0.18 |
| No change | 7 (9.6) | 0 (0.0) | 7 (13.5) |  |
| Mild change to one area | 14 (19.2) | 4 (19.1) | 10 (19.2) |  |
| Moderate (change in 2 areas) | 28 (38.4) | 7 (33.3) | 21 (40.4) |  |
| Severe (change in 3+ areas) | 24 (32.9) | 10 (47.6) | 14 (26.9) |  |
| **Family income / employment** |  |  |  | 0.32 |
| No change | 33 (45.2) | 8 (38.1) | 25 (48.1) |  |
| Mild, able to meet all needs and pay bills | 20 (27.4) | 5 (23.8) | 15 (28.9) |  |
| Moderate; having to make cuts but able to meet basic needs and pay bills | 14 (19.2) | 7 (33.3) | 7 (13.5) |  |
| Severe; unable to meet basic needs and/or pay bills | 6 (8.2) | 1 (4.8) | 5 (9.6) |  |
| **Food access** |  |  |  | 0.01 |
| No change | 52 (71.2) | 11 (52.4) | 41 (78.9) |  |
| Mild; enough food but difficulty getting to stores and/or finding needed items | 12 (16.4) | 8 (38.1) | 4 (7.7) |  |
| Moderate; occ without enough food and/or good quality foods | 9 (12.3) | 2 (9.5) | 7 (13.5) |  |
| Severe; frequently wo enough food and/or good quality foods | 0 (0.0) | 0 (0.0) | 0 (0.0) |  |
| **Medical Health Care Access** |  |  |  | 0.40 |
| No change | 42 (57.5) | 11 (52.4) | 31 (59.6) |  |
| Mild; appoints moved to telehealth | 18 (24.7) | 4 (19.1) | 14 (26.9) |  |
| Mod; delays or cancellations in appts and/or delays in getting Rx; minimal impact on health | 11 (15.1) | 5 (23.8) | 6 (11.5) |  |
| Severe; unable to access needed care resulting in severe risk and/or significant impact | 2 (2.7) | 1 (4.8) | 1 (1.9) |  |
| **Mental health treatment access** |  |  |  | 0.002 |
| No change | 53 (72.6) | 11 (52.4) | 42 (80.8) |  |
| Mild; appoints moved to telehealth | 13 (17.8) | 4 (19.1) | 9 (17.3) |  |
| Mod; delays or cancellations in appts and/or delays in getting Rx; minimal impact on health | 4 (5.5) | 4 (19.1) | 0 (0.0) |  |
| Severe; unable to access needed care resulting in severe risk and/or significant impact | 3 (4.1) | 2 (9.5) | 1 (1.9) |  |
| **Access to extended family and non-family social supports** |  |  |  | 0.08 |
| No change | 33 (45.2) | 6 (28.6) | 27 (51.9) |  |
| Mild, continued visits with social distancing and/or reg phone calls and/or televideo or social media contacts | 23 (31.5) | 7 (33.3) | 16 (30.8) |  |
| Moderate; loss of in person and remote contact with a few people, but not all supports | 15 (20.6) | 8 (38.1) | 7 (13.5) |  |
| Severe; loss of in person and remote contact with all supports | 2 (2.7) | 0 (0.0) | 2 (3.9) |  |
| **Experiences of stress related to coronavirus pandemic** |  |  |  | 0.04 |
| No change | 12 (16.4) | 0 (0.0) | 12 (23.1) |  |
| Mild; Occasional worries and/or minor stress-related symptoms (e.g., feel a little anxious, sad, and/or angry; mild/rare trouble sleeping) | 29 (39.7) | 12 (57.1) | 17 (32.7) |  |
| Moderate; Frequent worries and/or moderate stress-related symptoms (e.g., feel moderately anxious, sad, and/or angry; moderate/occasional trouble sleeping) | 21 (28.8) | 5 (23.8) | 16 (30.8) |  |
| Severe; Persistent worries and/or severe stress-related symptoms (e.g., feel extremely anxious, sad, and/or angry; severe/frequent trouble sleeping) | 11 (15.1) | 4 (19.1) | 7 (13.5) |  |
| **Stress and discord in the family** |  |  |  | 0.23 |
| No change | 40 (54.8) | 9 (42.9) | 31 (59.6) |  |
| Mild; Family members occasionally short-tempered with one another; no physical violence | 25 (34.3) | 8 (38.1) | 17 (32.7) |  |
| Mod; Family members frequently short-tempered with one another; and/or children in the home getting in physical fights with one another | 7 (9.6) | 4 (19.1) | 3 (5.8) |  |
| Severe; Family members frequently short-tempered with one another and adults in the home throwing things at one another, and/or knocking over furniture, and/or hitting and/or harming one another | 1 (1.4) | 0 (0.0) | 1 (1.9) |  |
| **Personal diagnosis of coronavirus** |  |  |  | 0.13 |
| No change | 21 (29.2) | 4 (19.1) | 17 (33.3) |  |
| Mild Symptoms effectively managed at home | 42 (58.3) | 12 (57.1) | 30 (58.8) |  |
| Moderate Symptoms severe and required brief hospitalization | 8 (11.1) | 5 (23.8) | 3 (5.9) |  |
| Severe Symptoms severe and required ventilation. | 1 (1.4) | 0 (0.0) | 1 (2.0) |  |
| **Number of immediate family members diagnosed with coronavirus, n=71** | 3 (2-4), range 0-10 | 4 (2, 5), 0-10 | 3 (2, 4), 0-7 | 0.10 |
| **Rate the symptoms of the person who was most sick** |  |  |  | 0.03 |
| Mild Symptoms effectively managed at home | 29 (51.8) | 5 (29.4) | 24 (61.5) |  |
| Moderate Symptoms severe and required brief hospitalization | 22 (39.3) | 11 (64.7) | 11 (28.2) |  |
| Severe Symptoms severe and required ventilation | 5 (8.9) | 1 (5.9) | 4 (10.3) |  |
| Immediate family member died from coronavirus | 0 (0.0) | 0 (0.0) | 0 (0.0) |  |
| **Number of extended family member(s) and/or close friends diagnosed with coronavirus, n=66** | 5 (3, 12), range 0-100 | 10 (4, 15), 0-100 | 5 (2, 11), 0-50 | 0.14 |
| Mild Symptoms effectively managed at home | 41 (70.7) | 10 (62.5) | 31 (73.8) | 0.41 |
| Moderate Symptoms severe and required brief hospitalization | 12 (20.7) | 4 (25.0) | 8 (19.1) |  |
| Severe Symptoms severe and required ventilation | 1 (1.7) | 1 (6.3) | 0 (0.0) |  |
| Extended family member and/or close friend died of coronavirus | 4 (6.9) | 1 (6.3) | 3 (7.1) |  |

**Supplemental Table 9.** School and vaccination status.

| Variables | Overall  N=50 children > 5 y | Not recovered  N=19 (38.0%) | Recovered  N=31 (62.0%) | P-value |
| --- | --- | --- | --- | --- |
| **Back to school, n=50** |  |  |  | 0.10 |
| Yes, in-person | 40 (80.0) | 13 (68.4) | 27 (87.1) |  |
| Yes, home | 7 (14.0) | 4 (21.1) | 3 (9.7) |  |
| Yes, mixed | 2 (4.0) | 2 (10.5) | 0 (0.0) |  |
| Not in school prior/No | 1 (2.0) | 0 (0.0) | 1 (3.2) |  |
| **Individualized Educational Plan (new)** | 16 (27.6) | 9 (45.0) | 7 (18.4) | 0.06 |
| **Individualized Educational Plan prior to pandemic** | 17 (21.5) | 7 (30.4) | 10 (17.9) | 0.24 |
| **My child is vaccinated** | 30 (40.5) | 9 (42.9) | 21 (39.6) | 0.80 |
| **Plan to vaccinate** | 8 (18.2); unsure 20 (45.5) | 1 (8.3) | 7 (21.9) | 0.74 |
| **Child is in a vaccination trial** | 2 (2.7) | 0 (0.0) | 2 (3.8) | 1.00 |

**Supplemental Table 10**. Multivariable logistic regression for the association with unfavorable child health-related quality of life status.

| Variable | N | Univariate  Odds ratio | 95% Confidence Interval | p-value | Multivariable  Odds ratio | 95% Confidence Interval | p-value |
| --- | --- | --- | --- | --- | --- | --- | --- |
| Any new or persistent symptom | 79 | 0.98 | 0.27, 3.60 | 0.97 |  |  |  |
| Total Family health related quality of life score | 74 | 0.95 | 0.92, 0.98 | 0.003 | 0.94 | 0.91, 0.98 | 0.01 |
| Hospital length of stay | 79 | 0.998 | 0.91, 1.09 | 0.98 |  |  |  |
| Sex | 79 | 0.79 | 0.22, 2.88 | 0.72 |  |  |  |
| Age | 78 | 0.99 | 0.89, 1.11 | 0.91 |  |  |  |
| Race | 77 | 1.07 | 0.58, 1.96 | 0.83 |  |  |  |
| Ethnicity | 58 | perfect |  |  |  |  |  |
| Neurologic comorbidity | 79 | 4.12 | 1.15, 14.70 | 0.03 | 10.57 | 1.71, 65.16 | 0.01 |
| Non-neurologic comorbidity | 77 | 1.00 | 0.27, 3.77 | 1.00 |  |  |  |
| Any comorbidity | 79 | 7.42 | 0.91, 60.91 | 0.06 |  |  |  |
| Cerebrospinal fluid: white blood cell count | 7 | 0.99 | 0.90, 1.09 | 0.78 |  |  |  |
| Pediatric Index of Mortality | 35 | 0.83 | 0.60, 1.16 | 0.29 |  |  |  |
| Glasgow Coma Scale score, initial | 67 | 0.84 | 0.67, 1.04 | 0.11 |  |  |  |
| Pediatric Logistic Organ Dysfunction score | 35 | 0.94 | 0.79, 1.13 | 0.51 |  |  |  |
| Sodium | 71 | 1.09 | 0.92, 1.29 | 0.34 |  |  |  |
| Hemoglobin | 68 | 1.09 | 0.81, 1.47 | 0.56 |  |  |  |
| C-reactive protein | 47 | 0.90 | 0.77, 1.05 | 0.19 |  |  |  |
| Ferritin | 37 | 0.99 | 0.98, 1.00 | 0.10 |  |  |  |
| Procalcitonin | 27 | 0.04 | 0.0005, 26.97 | 0.33 |  |  |  |
| Fibrinogen | 31 | 1.00 | 0.99, 1.01 | 0.57 |  |  |  |
| Alanine transaminase | 57 | 0.99 | 0.97, 1.02 | 0.53 |  |  |  |
| Aspartate transaminase | 53 | 0.9995 | 0.99, 1.01 | 0.88 |  |  |  |
| Prothrombin | 31 | 0.92 | 0.60, 1.42 | 0.72 |  |  |  |
| Partial Thromboplastin Time | 37 | 0.99 | 0.90, 1.10 | 0.87 |  |  |  |
| International Normalized Ratio | 37 | 0.08 | 0.001, 49.84 | 0.44 |  |  |  |
| D-dimer | 32 | 0.48 | 0.15, 1.50 | 0.21 |  |  |  |
| Steroids | 79 | 0.78 | 0.19, 3.20 | 0.73 |  |  |  |
| Remdesivir | 79 | 3.22 | 0.80, 12.94 | 0.10 |  |  |  |
| Intravenous immune globulin | 63 | perfect |  |  |  |  |  |
| Any treatment | 79 | 0.96 | 0.26, 3.52 | 0.95 |  |  |  |
| Epoch | 79 | 1.50 | 0.52, 4.31 | 0.45 |  |  |  |
| COVID vs. MIS-C | 62 | perfect |  |  |  |  |  |
| Any neuromanifestation | 79 | 0.58 | 0.17, 2.01 | 0.39 |  |  |  |
| Severe neuromanifestation | 79 | 1.18 | 0.32, 4.35 | 0.81 |  |  |  |
| White blood cells | 68 | 1.00 | 0.999, 1.000 | 0.98 |  |  |  |
| Lymphocytes | 65 | 1.00 | 0.9996, 1.000 | 0.71 |  |  |  |
| Platelets | 67 | 1.00 | 0.9999, 1.000 | 0.84 |  |  |  |
| Impairment | 77 | 5.81 | 1.10, 30.82 | 0.04 |  |  |  |
| Intensive care unit vs. ward | 79 | 0.84 | 0.23, 3.08 | 0.79 |  |  |  |
| Coronavirus Impact Scale score | 73 | 1.16 | 1.01, 1.33 | 0.04 |  |  |  |

Ethnicity, COVID vs. MIS-C, and Intravenous immune globulin variables were omitted because of estimability.

MIS-C, multisystem syndrome in children

**Supplemental Table 11**. Baseline characteristics of patients whose parents participated or did not participate in Tier 2.

| **Variables** | **Participated, n=79** | **Did not participate, n=993** |
| --- | --- | --- |
| Age, y, median (IQR) | 6.5 (2.0, 13.0) | 9.0 (1.5, 14.1) |
| Female sex, n (%) | 30 (38.0) | 488 (49.1) |
| Race |  |  |
| American Indian/Alaska native | 0 (0) | 10 (1.0) |
| Asian | 4 (5.2) | 35 (3.6) |
| Black | 17 (22.1) | 278 (28.8) |
| Native Hawaiian or other Pacific Islander | 0 (0) | 5 (0.5) |
| White | 53 (68.8) | 494 (51.2) |
| Unknown/other | 3 (3.9) | 143 (14.8) |
| Acute COVID (vs. MISC) | 62 (78.5) | 852 (85.8) |
| Any comorbidity | 51 (64.6) | 580 (58.4) |
