# Supplementary material for: Post-discharge outcomes of hospitalized children diagnosed with acute SARS-CoV-2 or MIS-C
Source: Front Pediatr. 2024 Feb 12;12:1340385. doi: 10.3389/fped.2024.1340385 (PMC10895015; doi:10.3389/fped.2024.1340385)
Supplement: Supplementary file 2 [file Datasheet1.pdf]

Post-acute COVID Questionnaire

Thank you for participating in our study to better understand long term outcomes in children and families affected by COVID-19. Please answer each question on behalf of your child. Once you 'Submit' a form you cannot return to it.

This section is required

Progress: 0%

.

Study ID ________________________________

Child's current age?

- ◯ 1-12 months
- ◯ 13-24 months
- ◯ 2-4 years
- ◯ 5-7 years
- ◯ 8-12 years
- ◯ 13-18 years

Consent

Please complete the survey below.

Thank you!

---

3) Do you consent?

◯ Yes
◯ No
(Just testing consent conditional)

Symptoms

This section is required
Progress: 15%

.

For each of the symptoms listed below, please answer whether your child had any new or continuing symptoms after the hospitalization that included a COVID-19 or MIS-C diagnosis. You can type in other symptoms at the bottom of this section.

	Yes, new symptom that started after hospital discharge	Yes, symptom occurred in the hospital and continued after discharge	No	Unsure
Headache	○	○	○	○
Loss of smell	○	○	○	○
Loss of taste	○	○	○	○
Vision problems	○	○	○	○
Lightheadedness or fainting	○	○	○	○
Seizures	○	○	○	○
Numbness	○	○	○	○
Weakness	○	○	○	○
Shooting or burning pain	○	○	○	○
Tingling sensations	○	○	○	○
Muscle aches or pain	○	○	○	○
Dizziness (room is spinning)	○	○	○	○
Balance problems	○	○	○	○
Delirium or confusion	○	○	○	○
Trouble with remembering things	○	○	○	○
Thinking or concentration problem	○	○	○	○
Sad or depressed	○	○	○	○
Anxious	○	○	○	○
Fever	○	○	○	○
Cough	○	○	○	○
Fatigue or tiredness	○	○	○	○
Loss of appetite	○	○	○	○
Diarrhea	○	○	○	○
Throat Pain	○	○	○	○
Abdominal Pain	○	○	○	○

Swollen glands	◯	◯	◯	◯
Vomiting or nausea	◯	◯	◯	◯
Joint pain	◯	◯	◯	◯
Sleep problems	◯	◯	◯	◯
Palpitations (rapid heartbeat, skipping heartbeats)	◯	◯	◯	◯
Trouble breathing	◯	◯	◯	◯
Speaking or communicating problem	◯	◯	◯	◯
Swallowing or chewing problem	◯	◯	◯	◯

Other symptom #1, please describe __________________________________

New or Continuing symptom #1?
◯ New
◯ Continuing

Other symptom #2, please describe __________________________________

New or Continuing symptom #2?
◯ New
◯ Continuing

Other symptom #3, please describe __________________________________

New or Continuing symptom #3?
◯ New
◯ Continuing

Please check which symptom, if any, has had the most negative impact on your child's health and functioning after hospital discharge

- ◯ Headache
- ◯ Loss of smell
- ◯ Loss of taste
- ◯ Vision problems
- ◯ Lightheadedness or fainting
- ◯ Seizures
- ◯ Numbness
- ◯ Weakness
- ◯ Shooting or burning pain
- ◯ Tingling sensations
- ◯ Muscle aches or pain
- ◯ Dizziness (room is spinning)
- ◯ Balance problems
- ◯ Delirium or confusion
- ◯ Trouble with remembering things
- ◯ Thinking or concentration problems
- ◯ Sad or depressed
- ◯ Anxious
- ◯ Fever
- ◯ Cough
- ◯ Fatigue or tiredness
- ◯ Loss of appetite
- ◯ Diarrhea
- ◯ Throat Pain
- ◯ Abdominal Pain
- ◯ Swollen glands
- ◯ Vomiting or nausea
- ◯ Joint pain
- ◯ Sleep problems
- ◯ Palpitations (rapid heartbeat, skipping heartbeats)
- ◯ Trouble breathing
- ◯ Speaking or communicating problem
- ◯ Swallowing or chewing problem
- ◯ Other symptom #1
- ◯ Other symptom #2
- ◯ Other symptom #3
- ◯ None

Healthcare Utilization

The questions below relate to therapies and emergency and hospital readmissions your child experienced since the hospitalization that included COVID-19/MIS-C diagnosis.

This section is required

Progress: 35%

.

Since your child's recent hospitalization that included a COVID-19/MIS-C diagnosis, has your child been evaluated in an emergency department?	◯ Yes ◯ No
How many total emergency visits did your child have since that hospitalization?	__________________
Approximately how many weeks or months after hospital discharge did the first emergency department visit occur?	◯ 0-2 weeks<br>◯ 2-4 weeks<br>◯ 1-3months<br>◯ 3-6months<br>◯ >6 months
What was the reason he or she needed to be evaluated in the first emergency department visit after discharge from the hospital?	__________________
What was your child diagnosed with in the emergency department the first time he or she visited?	__________________
What was your child's disposition during this emergency department the first time he or she visited?	◯ Home with you<br>◯ Hospital intensive care unit<br>◯ Hospital ward<br>◯ Long term care facility<br>◯ Other
Other	__________________
Approximately how many weeks or months after hospital discharge did the most recent emergency department visit occur?	◯ 0-2 weeks<br>◯ 2-4 weeks<br>◯ 1-3months<br>◯ 3-6months<br>◯ >6 months
What was the reason he or she needed to be evaluated in the emergency department the most recent time he or she visited?	__________________
What was your child diagnosed with in the emergency department the most recent time he or she visited?	__________________

What was your child's disposition during this emergency department the most recent time he or she visited?	◯ Home with you<br>◯ Hospital intensive care unit<br>◯ Hospital ward<br>◯ Long term care facility<br>◯ Other
Other	__________________________________
Since your child's recent hospitalization that included a COVID-19/MIS-C diagnosis, has your child stayed overnight in a hospital?	◯ Yes ◯ No
How many total hospital re-admissions did your child have?	____________________________
Approximately how many weeks or months after hospital discharge did the first hospital readmission occur?	◯ 0-2 weeks<br>◯ 2-4 weeks<br>◯ 1-3months<br>◯ 3-6months<br>◯ >6 months
What was your child diagnosed with during this hospitalization?	__________________________________
Was your child ever admitted to an intensive care unit during this visit?	◯ Yes ◯ No
What was your child's disposition during this hospital admission?	◯ Home with you<br>◯ Inpatient rehabilitation<br>◯ Long term care facility<br>◯ Other
Other	__________________________________
Approximately how many weeks or months after hospital discharge did the most recent hospital readmission occur?	◯ 0-2 weeks<br>◯ 2-4 weeks<br>◯ 1-3months<br>◯ 3-6months<br>◯ >6 months
What was your child diagnosed with during this hospitalization?	__________________________________
Was your child ever admitted to an intensive care unit during this visit?	◯ Yes ◯ No
What was your child's disposition during this hospital admission?	◯ Home with you<br>◯ Hospital intensive care unit<br>◯ Hospital ward<br>◯ Long term care facility<br>◯ Other

Other

____

After discharge from the hospital where your child was positive for COVID-19 or MIS-C, did your child receive care from any of the following health experts and/or clinics:

Physical therapy provider	◯ Yes ◯ No
Occupational therapy provider	◯ Yes ◯ No
Speech and Language therapy provider	◯ Yes ◯ No
Primary care provider	◯ Yes ◯ No
Behavioral Health/Psychiatry/Psychology specialist	◯ Yes ◯ No
Neuropsychologist	◯ Yes ◯ No
Neurology specialist	◯ Yes ◯ No
Rehabilitation specialist	◯ Yes ◯ No
Pulmonary (lung) specialist	◯ Yes ◯ No
Cardiology (heart) specialist	◯ Yes ◯ No
Post-Intensive Care clinic	◯ Yes ◯ No
Post-COVID clinic	◯ Yes ◯ No
Other health service expert or clinic:	◯ Yes ◯ No

Please specify

____

Medications

This section is optional

Progress: 40%

.

82) Is your child taking any new medications after discharge from the hospitalization from COVID-19 for any of the following treatment reasons (by category)?

- ☐ Seizures
- ☐ Pain
- ☐ Anxiety
- ☐ Depression
- ☐ Drug withdrawal
- ☐ Heart problems
- ☐ Lung problems
- ☐ Antibiotics
- ☐ Steroids
- ☐ Other psychiatric medications (e.g., mood stabilizers, antipsychotics)
- ☐ Postural orthostatic tachycardia syndrome (POTS)

83) Other medications

____

Participation

This section is required

Progress: 45%

.

Do you feel your child has fully recovered from his or her COVID-19 or MIS-C related illness?	◯ Yes<br>◯ No<br>◯ Unsure
Has your child returned to school since being discharged from the hospital in which he or she had COVID-19 or MIS-C related illness?	◯ Yes in person<br>◯ Yes from home<br>◯ Yes mixed<br>◯ Not in school prior to COVID<br>◯ No<br>◯ Not enrolled in school
If yes (returned to school), is your child receiving any special education services or accommodations? This means any added services that help your child learn and are provided by the school.	◯ Yes ◯ No
Was your child receiving special educational services or resources (for example a 504 plan or Individualized Education Plan) when attending school prior to developing COVID-19 or MIS-C?	◯ Yes ◯ No
Does your child have any new problems since the hospital admission that are not being treated or managed?	◯ Yes<br>◯ No
If yes, please describe the problem(s) and the reason(s) your child's new problem is not being addressed.	__________________________________
Please tell us about any other ways your child's hospitalization for COVID-19 or MISC-C has impacted your life.	__________________________________

Functional Status Scale measure

Please answer the questions below about your child’s function in 6 domains.

This section is required

Progress: 55%

.

Please choose the answer that best describes your child's mental status or alertness at baseline.	◯ Normal sleep/wake periods; appropriate responsiveness.<br>◯ Sleepy but arousable to noise/touch/movement and/or periods of social nonresponsiveness.<br>◯ Lethargic and/or irritable.<br>◯ Minimal arousal to stimuli (stupor).<br>◯ Unresponsive, coma, and/or vegetative state.
Please choose the answer that best describes your child's sensory functioning status (e.g., hearing, vision, response to touch).	◯ Intact hearing and vision and responsive to touch.<br>◯ Suspected hearing or vision loss.<br>◯ Not reactive to auditory stimuli 'or' to visual stimuli.<br>◯ Not reactive to auditory stimuli 'and' to visual stimuli<br>◯ Abnormal responses to pain or touch.
Please choose the answer that best describes your child's communication status.	◯ Appropriate noncrying vocalization, interactive facial expressiveness, or gestures.<br>◯ Diminished vocalization, facial expression, and/or social responsiveness.<br>◯ Absence of attention getting behavior.<br>◯ No demonstration of discomfort.<br>◯ Absence of communication.
Please choose the answer that best describes your child's physical functioning status.	◯ Coordinated body movements, normal muscle control, and awareness of action and reason.<br>◯ One limb functionally impaired.<br>◯ Two limbs functionally impaired.<br>◯ Poor head control.<br>◯ Diffuse spasticity, paralysis, or decerebrate/decorticate posturing.
Please choose the answer that best describes your child's feeding status.	◯ All food taken by mouth with age-appropriate help.<br>◯ Nothing by mouth or need for age-appropriate help with feeding.<br>◯ Oral and tube feedings.<br>◯ Parenteral nutrition with oral or tube feedings.<br>◯ All parenteral nutrition.
Please choose the answer that best describes your child's respiratory (breathing) status.	◯ Room air and no artificial support or aids.<br>◯ Oxygen/treatment and/or suctioning.<br>◯ Tracheostomy.<br>◯ Continuous positive airway pressure treatment for all or part of the day and/ or mechanical ventilator support for part of the day.<br>◯ Mechanical ventilator support for all of the day and night.
Functional Status Scale (FSS) Total	______________________________

Pediatric Quality of Life Inventory™ (PedsQL™) Parent Report 1-12 Months

This section is required
Progress: 70%

.

DIRECTIONS

On the following page is a list of things that might be a problem for your child.
Please tell us how much of a problem each one has been for your child during the past ONE month by choosing:

0 if it is never a problem
1 if it is almost never a problem
2 if it is sometimes a problem
3 if it is often a problem
4 if it is almost always a problem

There are no right or wrong answers.

In the past ONE month, how much of a problem has your child had with ...

PHYSICAL FUNCTIONING (problems with...)

		Never0	Almost Never1	Sometimes2	Often3	Almost Always4
98)	1. Low energy level	○	○	○	○	○
99)	2. Difficulty participating in active play	○	○	○	○	○
100)	3. Having hurts or aches	○	○	○	○	○
101)	4. Feeling tired	○	○	○	○	○
102)	5. Being lethargic	○	○	○	○	○
103)	6. Resting a lot	○	○	○	○	○

104) Scaled Score

____

PHYSICAL SYMPTOMS (problems with...)

	Never0	Almost Never1	Sometimes2	Often3	Almost Always4
105) 1. Having gas	◯	◯	◯	◯	◯
106) 2. Spitting up after eating	◯	◯	◯	◯	◯
107) 3. Difficulty breathing	◯	◯	◯	◯	◯
108) 4. Being sick to his/her stomach	◯	◯	◯	◯	◯
109) 5. Difficulty swallowing	◯	◯	◯	◯	◯
110) 6. Being constipated	◯	◯	◯	◯	◯
111) 7. Having a rash	◯	◯	◯	◯	◯
112) 8. Having diarrhea	◯	◯	◯	◯	◯
113) 9. Wheezing	◯	◯	◯	◯	◯
114) 10. Vomiting	◯	◯	◯	◯	◯

115) Scaled Score

____

EMOTIONAL FUNCTIONING (problems with...)

	Never0	Almost Never1	Sometimes2	Often3	Almost Always4
116) 1. Feeling afraid or scared	◯	◯	◯	◯	◯
117) 2. Feeling angry	◯	◯	◯	◯	◯
118) 3. Crying or fussing when left alone	◯	◯	◯	◯	◯
119) 4. Difficulty soothing himself/herself when upset	◯	◯	◯	◯	◯
120) 5. Difficulty falling asleep	◯	◯	◯	◯	◯
121) 6. Crying or fussing while being cuddled	◯	◯	◯	◯	◯
122) 7. Feeling sad	◯	◯	◯	◯	◯
123) 8. Difficulty being soothed when picked up or held	◯	◯	◯	◯	◯
124) 9. Difficulty sleeping mostly through the night	◯	◯	◯	◯	◯
125) 10. Crying a lot	◯	◯	◯	◯	◯
126) 11. Feeling cranky	◯	◯	◯	◯	◯
127) 12. Difficulty taking naps during the day	◯	◯	◯	◯	◯

128) Scaled Score

____

Social Functioning (problems with.....)

	Never0	Almost Never1	Sometimes2	Often3	Almost Always4
129) 1. Not smiling at others	◯	◯	◯	◯	◯
130) 2. Not laughing when tickled	◯	◯	◯	◯	◯
131) 3. Not making eye contact with a caregiver	◯	◯	◯	◯	◯
132) 4. Not laughing when cuddled	◯	◯	◯	◯	◯

133) Scaled Score ______________________

COGNITIVE FUNCTIONING (problems with...)

	Never0	Almost Never1	Sometimes2	Often3	Almost Always4
134) 1. Not imitating caregivers' actions	◯	◯	◯	◯	◯
135) 2. Not imitating caregivers' facial expressions	◯	◯	◯	◯	◯
136) 3. Not imitating caregivers' sounds	◯	◯	◯	◯	◯
137) 4. Not able to fix his/her attention on objects	◯	◯	◯	◯	◯

138) Scaled Score ______________________

139) Physical Health ______________________

140) Psychosocial ______________________

141) Total Scale ______________________

Pediatric Quality of Life Inventory™ (PedsQL™) Parent Report 13-24 Months

This section is required
Progress: 70%

.

DIRECTIONS

On the following page is a list of things that might be a problem for your child.
Please tell us how much of a problem each one has been for your child during the past ONE month by choosing:

0 if it is never a problem
1 if it is almost never a problem
2 if it is sometimes a problem
3 if it is often a problem
4 if it is almost always a problem

There are no right or wrong answers.

In the past ONE month, how much of a problem has your child had with ...

PHYSICAL FUNCTIONING (problems with...)

	Never0	Almost Never1	Sometimes2	Often3	Almost Always4
142) 1. Low energy level	◯	◯	◯	◯	◯
143) 2. Difficulty participating in active play	◯	◯	◯	◯	◯
144) 3. Having hurts or aches	◯	◯	◯	◯	◯
145) 4. Feeling tired	◯	◯	◯	◯	◯
146) 5. Being lethargic	◯	◯	◯	◯	◯
147) 6. Resting a lot	◯	◯	◯	◯	◯
148) 7. Feeling too tired to play	◯	◯	◯	◯	◯
149) 8. Difficulty walking	◯	◯	◯	◯	◯
150) 9. Difficulty running a short distance without falling	◯	◯	◯	◯	◯

151) Scaled Score ______________________

PHYSICAL SYMPTOMS (problems with...)

	Never0	Almost Never1	Sometimes2	Often3	Almost Always4
152) 1. Having gas	○	○	○	○	○
153) 2. Spitting up after eating	○	○	○	○	○
154) 3. Difficulty breathing	○	○	○	○	○
155) 4. Being sick to his/her stomach	○	○	○	○	○
156) 5. Difficulty swallowing	○	○	○	○	○
157) 6. Being constipated	○	○	○	○	○
158) 7. Having a rash	○	○	○	○	○
159) 8. Having diarrhea	○	○	○	○	○
160) 9. Wheezing	○	○	○	○	○
161) 10. Vomiting	○	○	○	○	○

162) Scaled Score

____

EMOTIONAL FUNCTIONING (problems with...)

	Never0	Almost Never1	Sometimes2	Often3	Almost Always4
163) 1. Feeling afraid or scared	○	○	○	○	○
164) 2. Feeling angry	○	○	○	○	○
165) 3. Crying or fussing when left alone	○	○	○	○	○
166) 4. Difficulty soothing himself/herself when upset	○	○	○	○	○
167) 5. Difficulty falling asleep	○	○	○	○	○
168) 6. Crying or fussing while being cuddled	○	○	○	○	○
169) 7. Feeling sad	○	○	○	○	○
170) 8. Difficulty being soothed when picked up or held	○	○	○	○	○
171) 9. Difficulty sleeping mostly through the night	○	○	○	○	○
172) 10. Crying a lot	○	○	○	○	○
173) 11. Feeling cranky	○	○	○	○	○
174) 12. Difficulty taking naps during the day	○	○	○	○	○

175) Scaled Score

____

SOCIAL FUNCTIONING (problems with.....)

	Never0	Almost Never1	Sometimes2	Often3	Almost Always4
176) 1. Not smiling at others	○	○	○	○	○
177) 2. Not laughing when tickled	○	○	○	○	○
178) 3. Not making eye contact with a caregiver	○	○	○	○	○
179) 4. Not laughing when cuddled	○	○	○	○	○
180) 5. Being uncomfortable around other children	○	○	○	○	○

181) Scaled Score

____

COGNITIVE FUNCTIONING (problems with...)

	Never0	Almost Never1	Sometimes2	Often3	Almost Always4
182) 1. Not imitating caregivers' actions	○	○	○	○	○
183) 2. Not imitating caregivers' facial expressions	○	○	○	○	○
184) 3. Not imitating caregivers' sounds	○	○	○	○	○
185) 4. Not able to fix his/her attention on objects	○	○	○	○	○
186) 5. Not imitating caregivers' speech	○	○	○	○	○
187) 6. Difficulty pointing to his/her body parts when asked	○	○	○	○	○
188) 7. Difficulty naming familiar objects	○	○	○	○	○
189) 8. Difficulty repeating words	○	○	○	○	○
190) 9. Difficulty keeping his/her attention on things	○	○	○	○	○

191) Scaled Score

____

192) Physical Health

____

193) Psychosocial

____

194) Total Scale

____

Pediatric Quality of Life Inventory™ (PedsQL™) Parent Report 2-4 years

This section is required
Progress: 70%

.

On this page, there is a list of things that might be a problem for your child. Please tell us how much of a problem each one has been for your child during the past ONE month by checking:

0 if it is never a problem
1 if it is almost never a problem
2 if it is sometimes a problem
3 if it is often a problem
4 if it is almost always a problem

There are no right or wrong answers.
If you do not understand a question, please ask for help

In the past one month, how much of a problem has your child had with...

Physical Functioning (problems with...)

	Never0	Almost Never1	Sometimes2	Often3	Almost Always4
195) 1. Walking	○	○	○	○	○
196) 2. Running	○	○	○	○	○
197) 3. Participating in active play or exercise	○	○	○	○	○
198) 4. Lifting something heavy	○	○	○	○	○
199) 5. Bathing	○	○	○	○	○
200) 6. Helping to pick up his or her toys	○	○	○	○	○
201) 7. Having hurts or aches	○	○	○	○	○
202) 8. Low energy level	○	○	○	○	○

203) Scaled Score

____

Emotional Functioning (problems with?)

	Never0	Almost Never1	Sometimes2	Often3	Almost Always4
204) 1. Feeling afraid or scared	○	○	○	○	○
205) 2. Feeling sad or blue	○	○	○	○	○
206) 3. Feeling angry	○	○	○	○	○
207) 4. Trouble sleeping	○	○	○	○	○
208) 5. Worrying	○	○	○	○	○

209) Scaled Score

____

Social Functioning (problems with)

	Never0	Almost Never1	Sometimes2	Often3	Almost Always4
210) 1. Playing with other children	○	○	○	○	○
211) 2. Other kids not wanting to play with him or her	○	○	○	○	○
212) 3. Getting teased by other children	○	○	○	○	○
213) 4. Not able to do things that other children his or her age can do	○	○	○	○	○
214) 5. Keeping up when playing with other children	○	○	○	○	○

215) Scaled Score

____

School Functioning (problems with)

	Never0	Almost Never1	Sometimes2	Often3	Almost Always4	N/A, Child does not attend school or daycare-1
216) 1. Doing the same school activities as peers	○	○	○	○	○	○
217) 2. Missing school/daycare because of not feeling well	○	○	○	○	○	○
218) 3. Missing school/daycare to go to the doctor or hospital	○	○	○	○	○	○

219) Scaled Score

____

Cognitive Fatigue

	Never0	Almost Never1	Sometimes2	Often3	Almost Always4
220) Difficulty keeping his/her attention on things	○	○	○	○	○
221) Difficulty remembering what people tell him/her	○	○	○	○	○
222) Difficulty remembering what he/she just heard	○	○	○	○	○
223) Difficulty thinking quickly	○	○	○	○	○
224) Trouble remembering what he/she was just thinking	○	○	○	○	○
225) Trouble remembering more than one thing at a time	○	○	○	○	○

226) Scaled Score

____

227) Psychosocial

____

228) Total Scale

____

Pediatric Quality of Life Inventory™ (PedsQL™) Parent Report 5-7 years

This section is required
Progress: 70%

.

DIRECTIONS

On the following page is a list of things that might be a problem for your child.
Please tell us how much of a problem each one has been for your child during the past ONE month by choosing:

0 if it is never a problem
1 if it is almost never a problem
2 if it is sometimes a problem
3 if it is often a problem
4 if it is almost always a problem

There are no right or wrong answers.
If you do not understand a question, please ask for help.

In the past one month, how much of a problem has your child had with...

Physical Functioning (problems with...)

	Never0	Almost Never1	Sometimes2	Often3	Almost Always4
229) 1. Walking more than one block	○	○	○	○	○
230) 2. Running	○	○	○	○	○
231) 3. Participating in sports activity or exercise	○	○	○	○	○
232) 4. Lifting something heavy	○	○	○	○	○
233) 5. Taking a bath or shower by him or herself	○	○	○	○	○
234) 6. Doing chores, like picking up his or her toys	○	○	○	○	○
235) 7. Having hurts or aches	○	○	○	○	○
236) 8. Low energy level	○	○	○	○	○

237) Scaled Score

____

Emotional Functioning (problems with....)

	Never0	Almost Never1	Sometimes2	Often3	Almost Always4
238) 1. Feeling afraid or scared	◯	◯	◯	◯	◯
239) 2. Feeling sad or blue	◯	◯	◯	◯	◯
240) 3. Feeling angry	◯	◯	◯	◯	◯
241) 4. Trouble sleeping	◯	◯	◯	◯	◯
242) 5. Worrying about what will happen to him or her	◯	◯	◯	◯	◯

243) Scaled Score

____

Social Functioning (problems with....)

	Never0	Almost Never1	Sometimes2	Often3	Almost Always4
244) 1. Getting along with other children	◯	◯	◯	◯	◯
245) 2. Other kids not wanting to be his or her friend	◯	◯	◯	◯	◯
246) 3. Getting teased by other children	◯	◯	◯	◯	◯
247) 4. Not able to do things that other children his or her age can do	◯	◯	◯	◯	◯
248) 5. Keeping up when playing with other children	◯	◯	◯	◯	◯

249) Scaled Score

____

School Functioning (problems with....)

	Never0	Almost Never1	Sometimes2	Often3	Almost Always4	N/A, Child does not attend school or daycare-1
250) 1. Paying attention in class	◯	◯	◯	◯	◯	◯
251) 2. Forgetting things	◯	◯	◯	◯	◯	◯
252) 3. Keeping up with school activities	◯	◯	◯	◯	◯	◯
253) 4. Missing school because of not feeling well	◯	◯	◯	◯	◯	◯
254) 5. Missing school to go to the doctor or hospital	◯	◯	◯	◯	◯	◯

255) Scaled Score

____

Cognitive Fatigue (Problems with ....)

	Never0	Almost Never1	Sometimes2	Often3	Almost Always4
256) Difficulty keeping his/her attention on things	○	○	○	○	○
257) Difficulty remembering what people tell him/her	○	○	○	○	○
258) Difficulty remembering what he/she just heard	○	○	○	○	○
259) Difficulty thinking quickly	○	○	○	○	○
260) Trouble remembering what he/she was just thinking	○	○	○	○	○
261) Trouble remembering more than one thing at a time	○	○	○	○	○

262) Scaled Score

____

263) Psychosocial

____

264) Total Scale

____

Pediatric Quality of Life Inventory™ (PedsQL™) Parent Report 8-12 years

This section is required
Progress: 70%

.

DIRECTIONS

On the following page is a list of things that might be a problem for your child.
Please tell us how much of a problem each one has been for your child during the past ONE month by choosing:

0 if it is never a problem
1 if it is almost never a problem
2 if it is sometimes a problem
3 if it is often a problem
4 if it is almost always a problem

There are no right or wrong answers.
If you do not understand a question, please ask for help.

In the past ONE month, how much of a problem has your child had with ...

PHYSICAL FUNCTIONING (problems with...)

	Never0	Almost Never1	Sometimes2	Often3	Almost Always4
265) 1. Walking more than one block	○	○	○	○	○
266) 2. Running	○	○	○	○	○
267) 3. Participating in sports activity or exercise	○	○	○	○	○
268) 4. Lifting something heavy	○	○	○	○	○
269) 5. Taking a bath or shower by him or herself	○	○	○	○	○
270) 6. Doing chores around the house	○	○	○	○	○
271) 7. Having hurts or aches	○	○	○	○	○
272) 8. Low energy level	○	○	○	○	○

273) Scaled Score

____

EMOTIONAL FUNCTIONING (problems with...)

	Never0	Almost Never1	Sometimes2	Often3	Almost Always4
274) 1. Feeling afraid or scared	○	○	○	○	○
275) 2. Feeling sad or blue	○	○	○	○	○
276) 3. Feeling angry	○	○	○	○	○
277) 4. Trouble sleeping	○	○	○	○	○
278) 5. Worrying about what will happen to him or her	○	○	○	○	○

279) Scaled Score

____

SOCIAL FUNCTIONING (problems with...)

	Never0	Almost Never1	Sometimes2	Often3	Almost Always4
280) 1. Getting along with other children	○	○	○	○	○
281) 2. Other kids not wanting to be his or her friend	○	○	○	○	○
282) 3. Getting teased by other children	○	○	○	○	○
283) 4. Not able to do things that other children his or her age can do	○	○	○	○	○
284) 5. Keeping up when playing with other children	○	○	○	○	○

285) Scaled Score

____

SCHOOL FUNCTIONING (problems with...)

	Never0	Almost Never1	Sometimes2	Often3	Almost Always4
286) 1. Paying attention in class	○	○	○	○	○
287) 2. Forgetting things	○	○	○	○	○
288) 3. Keeping up with schoolwork	○	○	○	○	○
289) 4. Missing school because of not feeling well	○	○	○	○	○
290) 5. Missing school to go to the doctor or hospital	○	○	○	○	○

291) Scaled Score

____

Cognitive Fatigue (problems with...)

	Never0	Almost Never1	Sometimes2	Often3	Almost Always4
292) Difficulty keeping his/her attention on things	○	○	○	○	○
293) Difficulty remembering what people tell him/her	○	○	○	○	○
294) Difficulty remembering what he/she just heard	○	○	○	○	○
295) Difficulty thinking quickly	○	○	○	○	○
296) Trouble remembering what he/she was just thinking	○	○	○	○	○
297) Trouble remembering more than one thing at a time	○	○	○	○	○

298) Scaled Score

____

299) Psychosocial

____

300) Total Scale

____

Pediatric Quality of Life Inventory™ (PedsQL™) Parent Report 13-18 years

This section is required
Progress: 70%

.

DIRECTIONS

On this page, there is a list of things that might be a problem for your teen. Please tell us how much of a problem each one has been for your teen during the past ONE month by checking:

0 if it is never a problem
1 if it is almost never a problem
2 if it is sometimes a problem
3 if it is often a problem
4 if it is almost always a problem

There are no right or wrong answers.

In the past one month, how much of a problem has your teen had with...

Physical Functioning (problems with...)

		Never0	Almost Never1	Sometimes2	Often3	Almost Always4
301)	1. Walking more than one block	◯	◯	◯	◯	◯
302)	2. Running	◯	◯	◯	◯	◯
303)	3. Participating in sports activity or exercise	◯	◯	◯	◯	◯
304)	4. Lifting something heavy	◯	◯	◯	◯	◯
305)	5. Taking a bath or shower by him or herself	◯	◯	◯	◯	◯
306)	6. Doing chores around the house	◯	◯	◯	◯	◯
307)	7. Having hurts or aches	◯	◯	◯	◯	◯
308)	8. Low energy level	◯	◯	◯	◯	◯

309) Scaled Score ______________________

Emotional Functioning (problems with)

	Never0	Almost Never1	Sometimes2	Often3	Almost Always4
310) 1. Feeling afraid or scared	◯	◯	◯	◯	◯
311) 2. Feeling sad or blue	◯	◯	◯	◯	◯
312) 3. Feeling angry	◯	◯	◯	◯	◯
313) 4. Trouble sleeping	◯	◯	◯	◯	◯
314) 5. Worrying about what will happen to him or her	◯	◯	◯	◯	◯

315) Scaled Score

____

Social Functioning (problems with)

	Never0	Almost Never1	Sometimes2	Often3	Almost Always4
316) 1. Getting along with other teens	◯	◯	◯	◯	◯
317) 2. Other teens do not want to be his or her friend	◯	◯	◯	◯	◯
318) 3. Getting teased by other teens	◯	◯	◯	◯	◯
319) 4. Not able to do things that other teens his or her age can do	◯	◯	◯	◯	◯
320) 5. Keeping up with other teens	◯	◯	◯	◯	◯

321) Scaled Score

____

School Functioning (problems with)

	Never0	Almost Never1	Sometimes2	Often3	Almost Always4
322) 1. Paying attention in class	◯	◯	◯	◯	◯
323) 2. Forgetting things	◯	◯	◯	◯	◯
324) 3. Keeping up with schoolwork	◯	◯	◯	◯	◯
325) 4. Missing school because of not feeling well	◯	◯	◯	◯	◯
326) 5. Missing school to go to the doctor or hospital	◯	◯	◯	◯	◯

327) Scaled Score

____

Cognitive Fatigue (Problems with....)

	Never0	Almost Never1	Sometimes2	Often3	Almost Always4
328) Difficulty keeping his/her attention on things	○	○	○	○	○
329) Difficulty remembering what people tell him/her	○	○	○	○	○
330) Difficulty remembering what he/she just heard	○	○	○	○	○
331) Difficulty thinking quickly	○	○	○	○	○
332) Trouble remembering what he/she was just thinking	○	○	○	○	○
333) Trouble remembering more than one thing at a time	○	○	○	○	○

334) Scaled Score ______________________________

335) Psychosocial ______________________________

336) Total Scale ______________________________

PROMIS Global Health 7 + 2 Survey, Parent/Proxy

Please complete the survey below.

Thank you!

This section is required
Progress: 75%

.

Please respond to each question or statement by marking one box per row.

	Excellent	Very Good	Good	Fair	Poor
337) In general, would you say your child's health is:	◯	◯	◯	◯	◯
338) In general, would you say your child's quality of life is:	◯	◯	◯	◯	◯
339) In general, how would you rate your child's physical health?	◯	◯	◯	◯	◯
340) In general, how would you rate your child's mental health, including mood and ability to think?	◯	◯	◯	◯	◯

	Never	Rarely	Sometimes	Often	Always
341) In general, would you say your child's health is:	◯	◯	◯	◯	◯

	Always	Often	Sometimes	Rarely	Never
342) How often does your child have fun with friends?	◯	◯	◯	◯	◯
343) How often does your child feel that you listen to his or her ideas?	◯	◯	◯	◯	◯

In the past 7 days...

	Never	Almost Never	Sometimes	Often	Almost Always
344) My child got tired easily	◯	◯	◯	◯	◯
345) My child had trouble sleeping when he/she had pain	◯	◯	◯	◯	◯

---

346) Total (global 7 questions) __________________________

---

347) PROMIS Total: __________________________

Promis Young Child

This section is required
Progress: 75%

.

Please respond to each question or statement by marking one box per row.

	Excellent	Very Good	Good	Fair	Poor
348) In general, would you say your child's health is:	◯	◯	◯	◯	◯
349) In general, would you say your child's quality of life is:	◯	◯	◯	◯	◯
350) In general, how would you rate your child's physical health?	◯	◯	◯	◯	◯
351) In general, how would you rate your child's mental health?	◯	◯	◯	◯	◯
352) How would you rate your child's mood?	◯	◯	◯	◯	◯
353) How would you rate your child's social skills?	◯	◯	◯	◯	◯
354) How would you rate your child's ability to think?	◯	◯	◯	◯	◯
355) How well is your child meeting developmental milestones?	◯	◯	◯	◯	◯

Pediatric Cerebral Performance Category (PCPC) Scale

This section is optional
Progress: 78%

.

Please choose the category that best fits your child's functional status today.

- ◯ At age-appropriate level; if school-age child attending regular school classroom
- ◯ Conscious, alert, and able to interact at age-appropriate level; if school-age child attending regular school classroom, but grade perhaps not appropriate for age
- ◯ Conscious; sufficient cerebral function for age-appropriate independent activities of daily life; if schoolage child attending special education classroom and/or learning deficit present
- ◯ Conscious; dependent on others for daily support because of impaired brain function
- ◯ Any degree of coma, no interaction with environment

PedsQL™ Family Impact Module Version 2.0 survey

This section is required
Progress: 90%

.

Families of children sometimes have special concerns or difficulties because of the child's health. Below is a list of things that might be a problem for you. Please tell us how much of a problem each one has been for YOU during the past ONE month.

Physical

	Never	Almost Never	Sometimes	Often	Almost Always
357) Feel tired during the day	○	○	○	○	○
358) Feel tired when I wake up in the morning	○	○	○	○	○
359) Feel too tired to do the things I like to do	○	○	○	○	○
360) Get headaches	○	○	○	○	○
361) Feel physically weak	○	○	○	○	○
362) Feel sick to my stomach	○	○	○	○	○

363) Physical Score

____

Emotional

	Never	Almost Never	Sometimes	Often	Almost Always
364) Feel anxious	○	○	○	○	○
365) Feel sad	○	○	○	○	○
366) Feel angry	○	○	○	○	○
367) Feel frustrated	○	○	○	○	○
368) Feel helpless or hopeless	○	○	○	○	○

369) Emotional Score

____

Social

	Never	Almost Never	Sometimes	Often	Almost Always
370) Feel isolated from others	○	○	○	○	○
371) Have trouble getting support from others	○	○	○	○	○
372) Hard to find time for social activities	○	○	○	○	○

373)

	Never	Almost Never	Sometimes	Often	Almost Always
Do not have enough energy for social activities	○	○	○	○	○

374) Social Score

____

Cognitive

	Never	Almost Never	Sometimes	Often	Almost Always
375) It is hard for me to keep my attention on things	○	○	○	○	○
376) It is hard for me to remember what people tell me	○	○	○	○	○
377) It is hard for me to remember what I just heard	○	○	○	○	○
378) It is hard for me to think quickly	○	○	○	○	○
379) I have trouble remembering what I was just thinking	○	○	○	○	○

380) Cognitive Score

____

Communication

	Never	Almost Never	Sometimes	Often	Almost Always
381) I feel that others do not understand my family's situation	○	○	○	○	○
382) It is hard for me to talk about my child's health with others	○	○	○	○	○
383) It is hard for me to tell doctors and nurses how I feel	○	○	○	○	○

384) Communication Score

____

Worry

	Never	Almost Never	Sometimes	Often	Almost Always
385) I worry about whether or not my child's medical treatments are working	○	○	○	○	○
386) I worry about the side effects of my child's medications/medical treatments	○	○	○	○	○
387) I worry about how others will react to my child's condition	○	○	○	○	○

388)

	Never	Almost Never	Sometimes	Often	Almost Always
I worry about how my child's illness is affecting other family members	◯	◯	◯	◯	◯
389) I worry about my child's future	◯	◯	◯	◯	◯

390) Worry Score

____

Daily Activities

	Never	Almost Never	Sometimes	Often	Almost Always
391) Family activities taking more time and effort	◯	◯	◯	◯	◯
392) Difficulty finding time to finish household tasks	◯	◯	◯	◯	◯
393) Feeling too tired to finish household tasks	◯	◯	◯	◯	◯

394) Daily Activities Score

____

Family Relationships

	Never	Almost Never	Sometimes	Often	Almost Always
395) Lack of communication between family members	◯	◯	◯	◯	◯
396) Conflicts between family members	◯	◯	◯	◯	◯
397) Difficulty making decisions together as a family	◯	◯	◯	◯	◯
398) Difficulty solving family problems together	◯	◯	◯	◯	◯
399) Stress or tension between family members	◯	◯	◯	◯	◯

400) Family Relationships Score

____

401) Parent HRQL Summary Score

____

402) Family Functioning Summary Score

____

403) FIM Total Score

____

Coronavirus Impact Scale

This section is optional
Progress: 95%

.

Rate how much the Coronavirus pandemic has changed your life in each of the following ways: Routines	◯ No change.<br>◯ Mild. Change in only one area (e.g. work, education, social life, hobbies, religious activities).<br>◯ Moderate. Change in two areas (e.g. work, education, social life, hobbies, religious activities).<br>◯ Severe. Change in three or more areas (e.g. work, education, social life, hobbies, religious activities).
Family Income/employment	◯ No change.<br>◯ Mild. Small change; able to meet all needs and pay bills.<br>◯ Moderate. Having to make cuts but able to meet basic needs and pay bills.<br>◯ Severe. Unable to meet basic needs and/or pay bills.
Food access	◯ No change.<br>◯ Mild. Enough food but difficulty getting to stores and/or finding needed items.<br>◯ Moderate. Occasionally without enough food and/or good quality (e.g., healthy) foods.<br>◯ Severe. Frequently without enough food and/or good quality (e.g., healthy) foods.
Medical health care access	◯ No change.<br>◯ Mild. Appointments moved to telehealth.<br>◯ Moderate. Delays or cancellations in appointments and/or delays in getting prescriptions; changes have minimal impact on health.<br>◯ Severe. Unable to access needed care resulting in moderate to severe impact on health.
Mental health care access	◯ No change.<br>◯ Mild. Appointments moved to telehealth.<br>◯ Moderate. Delays or cancellations in appointments and/or delays in getting prescriptions; changes have minimal impact<br>◯ Severe. Unable to access needed care resulting in severe risk and/or significant impact
Access to extended family and on-family social supports	◯ No change.<br>◯ Mild. Continued visits with social distancing and/or regular phone calls and/or televideo or social media contacts.<br>◯ Moderate. Loss of in person and remote contact with a few people, but not all supports.<br>◯ Severe. Loss of in person and remote contact with all supports.

Experiences of stress related to coronavirus pandemic	◯ None.<br>◯ Mild. Occasional worries and/or minor stress-related symptoms (e.g., feel a little anxious, sad, and/or angry; mild/rare trouble sleeping).<br>◯ Moderate. Frequent worries and/or moderate stress-related symptoms (e.g., feel moderately anxious, sad, and/or angry; moderate/occasional trouble sleeping).<br>◯ Severe. Persistent worries and/or severe stress-related symptoms (e.g., feel extremely anxious, sad, and/or angry; severe/frequent trouble sleeping).
Stress and discord in the family	◯ None.<br>◯ Mild. Family members occasionally short-tempered with one another; no physical violence.<br>◯ Moderate. Family members frequently short-tempered with one another; and/or children in the home getting in physical fights with one another.<br>◯ Severe. Family members frequently short-tempered with one another and adults in the home throwing things at one another, and/or knocking over furniture, and/or hitting and/or harming one another.
Personal diagnosis of coronavirus	◯ None.<br>◯ Mild. Symptoms effectively managed at home.<br>◯ Moderate. Symptoms severe and required brief hospitalization.<br>◯ Severe. Symptoms severe and required ventilation.
Number of immediate family members diagnosed with coronavirus:	______________________________
Rate the symptoms of the person who was most sick:	◯ Mild. Symptoms effectively managed at home.<br>◯ Moderate. Symptoms severe and required brief hospitalization.<br>◯ Severe. Symptoms severe and required ventilation.<br>◯ Immediate family member died from coronavirus
Number of extended family member(s) and/or close friends diagnosed with coronavirus: ___	______________________________
Rate the symptoms of the person who was most sick:	◯ Mild. Symptoms effectively managed at home.<br>◯ Moderate. Symptoms severe and required brief hospitalization.<br>◯ Severe. Symptoms severe and required ventilation.<br>◯ Extended family member and/or close friend died of coronavirus.
Please tell us about any other ways the coronavirus pandemic has impacted your life:	_____________________________________

Vaccination

**Progress: 95%**<br><br>**.**	
Was your child vaccinated against COVID-19?	◯ Yes ◯ No
Do you plan to have your child vaccinated against COVID-19?	◯ Yes<br>◯ No<br>◯ Unsure
Did your child participate in a randomized trial to test COVID-19 vaccines?	◯ Yes ◯ No
If yes, and you are aware, did your child receive the vaccine or placebo (sugar)?	◯ Vaccine<br>◯ Placebo<br>◯ Unsure

THANK YOU

Thank you very much for completing the study. Please see the list below of resources for families with children affected by COVID and MIS-C.

1. Center for Disease Control (US-based)
Children, Teens, and Young Adults: Sections on school, coping and support, health and well-being in the pandemic.
https://www.cdc.gov/coronavirus/2019-ncov/daily-life-coping/caring-for-children.html

2. Local, national, and international post-COVID clinics. Your primary care provider, local or regional hospital, or Children's hospital may have resources for children affected by COVID/MIS-C.

One example is the Kennedy Krieger Institute has a Pediatric Post COVID-19 Rehabilitation Clinic:
https://www.kennedykrieger.org/patient-care/centers-and-programs/pediatric-post-covid-19-rehabilitation-clinic
